# Helios is critical for Treg effector differentiation in adipose autoimmunity

**DOI:** 10.1084/jem.20260870

**Published:** 2026-10-01

**Authors:** Angela M. Thornton, Xi Chen, Wenjia Cao, Tovah E. Markowitz, Kimmo Virtaneva, Paul A. Beare, Vinay Penna, Oksana Gavrilova, Jihyun Kim, Craig A. Martens, Jinfang Zhu, Ethan M. Shevach

**Affiliations:** 1 Cellular Immunology Section, Laboratory of Immune System Biology, National Institute of Allergy and Infectious Diseases, National Institutes of Health, Bethesda, MD, USA; 2 Molecular and Cellular Immunoregulation Section, Laboratory of Immune System Biology, National Institute of Allergy and Infectious Diseases, National Institutes of Health, Bethesda, MD, USA; 3 Integrated Data Sciences Section, Research Technologies Branch, National Institute of Allergy and Infectious Diseases, National Institutes of Health, Bethesda, MD, USA; 4 Genomic Research Section, Research Technologies Branch, National Institute of Allergy and Infectious Diseases, National Institutes of Health, Bethesda, MD, USA; 5 Mouse Metabolism Core, National Institute of Diabetes and Digestive and Kidney Diseases, National Institutes of Health, Bethesda, MD, USA

## Abstract

The deletion of the transcription factor Helios in regulatory T cells (Tregs) in mice leads to systemic Th1 immune activation, decreased T follicular regulatory cells, and enhanced germinal center formation. We report here these mice also exhibit acquired lipodystrophy, hepatic steatosis, and insulin resistance. Adipose tissue exhibited lymphocytic infiltration followed by CD8^+^ T cell–mediated destruction of adipocytes. Helios-deficient Tregs were more frequent in adipose tissue and showed an enhanced effector Treg (eTreg) phenotype (CD62L^lo^ Tcf1^lo^). The Helios-deficient eTregs in adipose tissue, but not spleen, failed to develop into terminally differentiated eTregs, which express ST2^+^, CXCR3^+^, IRF4, or Klrg1^+^. ChIP-seq studies demonstrated that Helios bound to regulatory elements in multiple genes (*Tcf7*, *Lef1*, *Bach2*, and *Id3*) known to restrict eTreg generation. Thus, Helios not only directly restricts eTreg generation but also indirectly enforces functional eTreg differentiation. In the absence of Helios, the process of eTreg differentiation proceeds unchecked, resulting in enhanced but dysfunctional eTregs, which fail to control adipose tissue homeostasis.

## Teaser

Thornton et al. demonstrate that in Tregs, the transcription factor Helios directly controls Tcf1, Lef1, Id3, and Bach2 to regulate effector Treg generation and terminal differentiation. Mice with Helios-deficient Tregs generate dysfunctional effector Tregs resulting in a unique adipose-specific autoimmunity.

## Introduction

CD4^+^Foxp3^+^ regulatory T cells (Tregs) suppress immune activation in a dominant manner and play a critical role in the maintenance of immune system homeostasis and self-tolerance (Rudensky, 2011; Shevach, 2011). Defects in Treg numbers, function, and stability have been proposed as major causative factors in both organ-specific and systemic models of autoimmunity. Paradoxically, many models of autoimmune disease are characterized by an increased frequency of Tregs, which express an “activated” phenotype, presumably, in an attempt to compensate for their dysfunction.

Tregs also control tissue homeostasis, and adipose tissue (AT) represents one of the best studied examples of this Treg function (Becker et al., 2017; Burzyn et al., 2013; Castoldi et al., 2015; Panduro et al., 2016). The percentage of Tregs of total CD4^+^ T cells is higher in AT compared with lymphoid tissues, and their transcriptome is distinct compared with splenic Tregs (Feuerer et al., 2009; Winer et al., 2009). In particular, AT Tregs are more highly activated, are clonally restricted, and secrete high levels of IL-10 (Feuerer et al., 2009; Han et al., 2014). Mice fed a high-fat diet have fewer AT Tregs, but treatment with anti-IL-2/IL-2R complexes to increase the number of Tregs resulted in lower blood glucose and increased insulin tolerance (Feuerer et al., 2009). In addition, leptin-deficient obese (*Ob/ob*) mice also have fewer Tregs but when administered anti-CD3 and β-glucosylceramide, exhibited increased Tregs with a concurrent decrease in macrophages and blood glucose levels (Ilan et al., 2010). Importantly, AT Tregs express the adipose-specific transcription factor (TF), PPARγ, required for their maintenance and identity in AT (Cipolletta et al., 2012), and they express the IL-33R (ST2) (Han et al., 2015; Kolodin et al., 2015; Vasanthakumar et al., 2015). The importance of the IL-33/ST2 axis in AT homeostasis has been demonstrated in many studies. *Ob/ob* mice treated with IL-33 have an increase in Th2 cells, a repolarization of M1 to M2 (CD206^+^) macrophages, and improved insulin sensitivity (Miller et al., 2010). High-fat diet mice treated with IL-33 exhibit increased Tregs, and increased ST2 on Tregs, with a concurrent decrease in macrophages, blood glucose, and insulin resistance (Brestoff et al., 2015; Han et al., 2015), while global deletion of either ST2 or IL-33 led to a marked decrease of Tregs in AT and increased insulin resistance (Molofsky et al., 2015; Vasanthakumar et al., 2015).

However, the intrinsic role of IL-33 signaling in Tregs has been questioned, as earlier studies were carried out in mice with germline deletions. Subsequently, one study in mice with a Treg-specific deletion of ST2 demonstrated that Tregs were, in fact, reduced in AT (Li et al., 2018), but Hemmers et al. did not note any differences in either number or phenotype of Tregs in the AT of mice with a Treg-specific deletion of ST2, calling into question the intrinsic role of IL-33 signaling in Treg maintenance in the adipose and highlighting the complex role that the ST2/IL-33 axis plays in adipose homeostasis (Hemmers et al., 2021).

Recent studies have demonstrated that Tregs are a heterogeneous population and can be divided into three major subpopulations: resting Tregs (rTregs), activated Tregs (aTregs), and effector Tregs (eTregs). rTregs are characterized as CD62^+^Tcf1^+^, aTregs as CD62^−^Tcf1^+^, and eTregs as CD62^−^TCF^−^ (Yang et al., 2019). eTregs may additionally express markers of terminal differentiation such as Klrg1, IRF4, and Batf (Sumida et al., 2024; Yang et al., 2019) and, analogous to effector CD4^+^ T cell subsets, can also be divided into T follicular regulatory cells (T_FR_), Th1-like, Th2-like, and Th17-like subsets, expressing the effector-specific TFs Bcl6, t-bet, Gata3, and RORγt, respectively. These eTreg subsets are present in peripheral lymphoid organs but are much more prevalent in tissue. In AT, two major eTreg subpopulations that have been described by Torres et al. are characterized by ST2 and CXCR3, respectively (Torres et al., 2024). In addition, Muñoz-Rojas et al. have used single-cell RNA sequencing (RNA-seq) to identify seven AT Treg subpopulations including an ST^+^ population, as well as t-bet^+^, 4-1BB^+^, IL-18R1^+^, and quiescent cells (Muñoz-Rojas et al., 2024). As differentiation to eTregs must be tightly controlled to avoid nonspecific immunosuppression, the TF Id3 blocks aTreg progression to eTregs and is critical for maintaining aTreg and rTreg homeostasis (Sullivan et al., 2019). In addition, the TFs Tcf1, Lef1, and Bach2 also block differentiation to effector subsets but promote T_FR_ differentiation (Sumida et al., 2024).

Treg-specific deletion of the TF, Helios, in mice results in generalized T cell activation, T follicular helper (T_FH_) expansion, and a systemic Th1 profile (Sebastian et al., 2016). We show here that in contrast to Scurfy mice or mice with a deletion of Foxp3 which develop inflammation in multiple organs, organ-specific inflammation in Helios knockout (KO) mice is uniquely limited to AT. In marked contrast to other models of Treg dysfunction in the AT that exhibit metabolic disruption, Helios-deficient mice develop acquired lipodystrophy with extensive T cell infiltrate and destruction of AT, leading to hepatic steatosis, markedly enlarged livers, and insulin resistance. Importantly, lipodystrophy is exclusively mediated by autoreactive CD8^+^ T cells that destroy AT through Fas-L– and cytotoxic-mediated mechanisms. Phenotypic analysis of the AT Tregs in these Helios-deficient mice demonstrates that they are skewed to the eTreg phenotype (CD62L^lo^ and Tcf1^−^) but fail to express markers of terminally differentiated eTregs such as CXCR3, ST2, and Klrg1. Chromatin immunoprecipitation sequencing (ChIP-seq) studies demonstrate that Helios binds to sites in the *Id3*, *Tcf7*, *Lef1*, and *Bach2* loci. As these genes collectively restrain eTreg development, our studies show that in the absence of Helios, the process of eTreg differentiation proceeds unchecked, resulting in enhanced but dysfunctional eTreg production. Thus, Helios is required both to maintain the aTreg pool and to generate proper terminally differentiated eTregs.

## Results

### Mice with Helios-deficient Tregs develop CD8^+^ T cell–mediated acquired lipodystrophy

**Figure 1.**
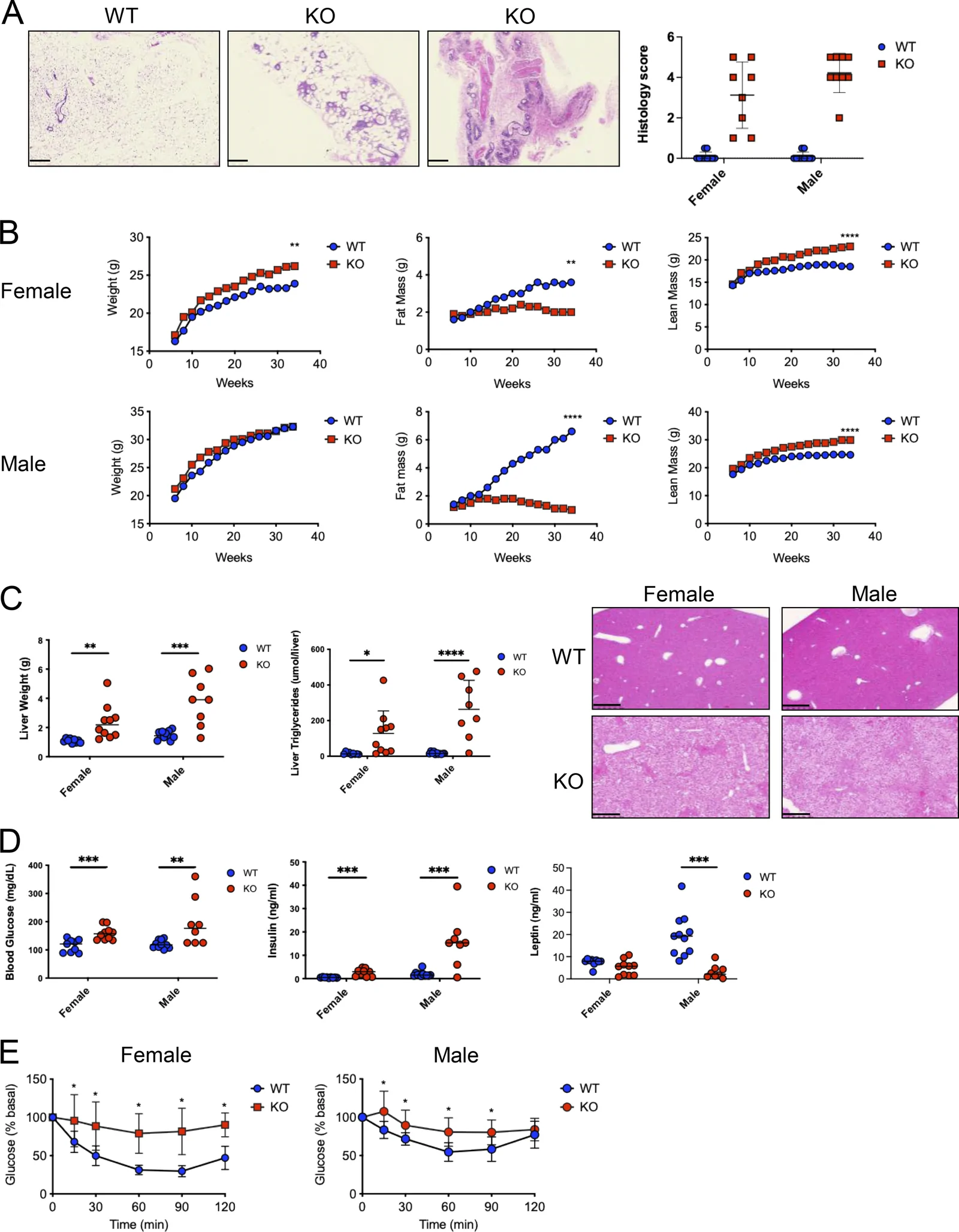
**Mice with Helios-deficient Tregs develop acquired lipodystrophy and metabolic dysregulation. (A)** Representative H&E staining of epididymal WAT from 3-mo-old WT and KO male mice and (right) histological scoring of infiltrate and AT loss in 4-mo-old WT and KO mice (scale 1–5). Scale bars represent 500 μm. **(B)** Body weight, fat mass, and lean mass were measured in female and male WT and KO mice every 2 wk until 36 wk old. **(C)** Livers from female and male WT and KO mice were weighed, liver triglycerides measured and samples fixed and analyzed by H&E staining at the end of the study period. Representative H&E stains are shown. Scale bars represent 500 μm. **(D)** Blood glucose, insulin, and leptin levels were measured. Blood was collected from the tail vein of nonfasted, 35-wk-old mice at euthanasia (36 wk). One sample from the female insulin KO group was excluded as it was >3 stdev of the mean from the rest of the group. **(E)** At 36 wk of age, female and male WT and KO mice were fasted overnight, given insulin i.v., and monitored for blood glucose levels at the indicated time points. For all experiments, WT female, *n* = 8; WT male, *n* = 11; KO female, *n* = 10; KO male, *n* = 8; data are plotted as the percentage of basal glucose. *P < 0.05, **P < 0.01, ***P < 0.001, ****P < 0.0001 by unpaired *t* tests.

**Figure S1.**
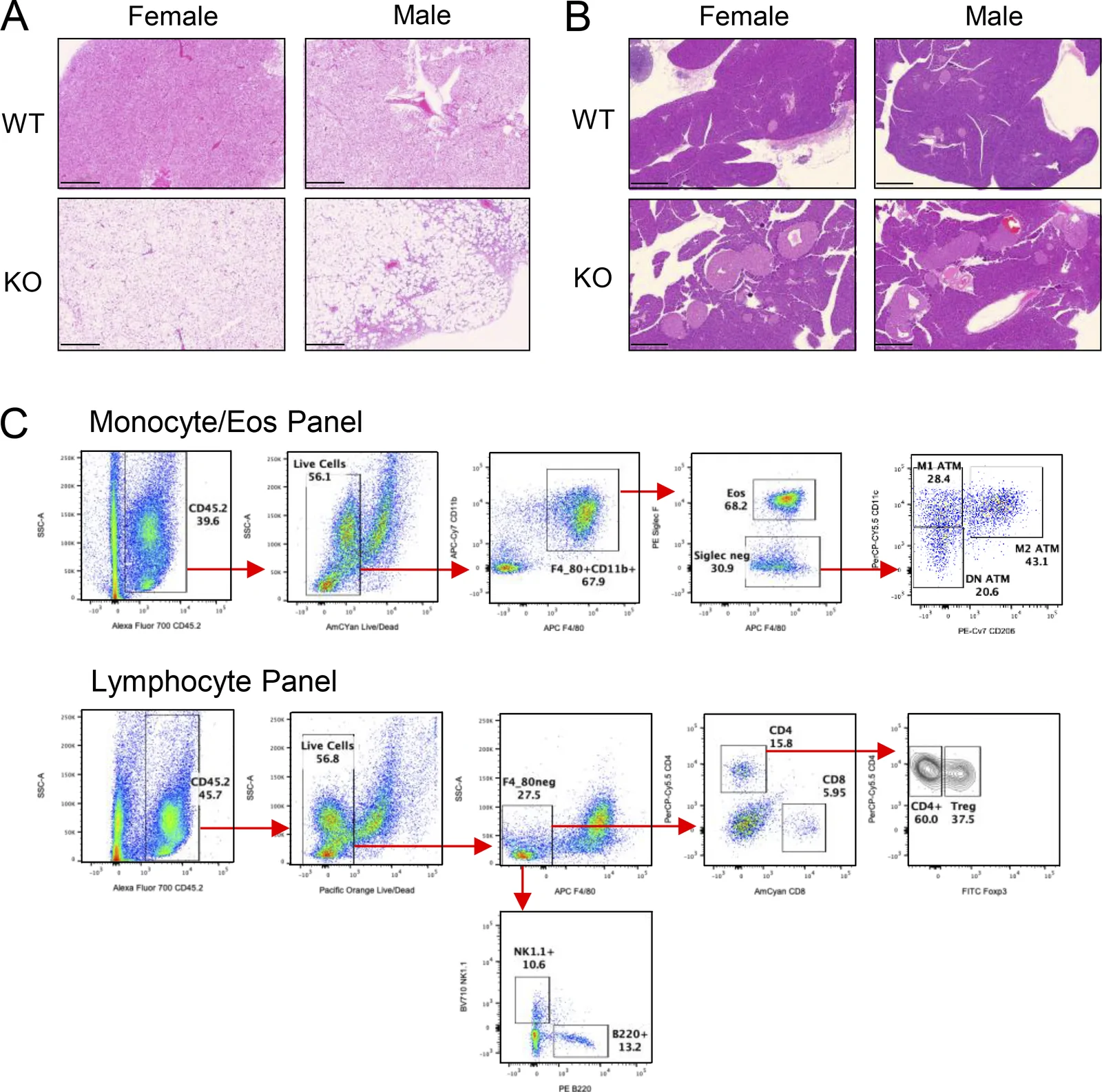
**Mice with Helios-deficient Tregs develop metabolic dysregulation. (A and B)** H&E staining of BAT (A) and pancreas (B) from 36-wk-old female and male WT and KO mice. Scale bars represent 500 μm. **(C)** Gating strategy for AT immunophenotyping.

We have previously published that mice with a Treg-specific deletion of the TF Helios (*Ikzf2*^fl/fl^ x Foxp3-Cre mice, hereafter KO) develop systemic immune activation with splenomegaly and lymphadenopathy, activation of T cells with a Th1 profile, and an extreme expansion of T_FH_ cells (Sebastian et al., 2016). KO mice also exhibit a loss of AT, an enlargement of the liver, and hepatic steatosis. To examine the loss of AT more thoroughly in KO mice, a full metabolic study was performed beginning at 4 wk of age. Our previous study showed that at 6 mo of age, KO mice had a complete loss of AT. At 3 mo, AT showed varying degrees of hematopoietic cell infiltrate, ranging from mild to moderate infiltration, to an almost complete destruction of white AT (WAT) (Fig. 1 A). H&E analysis revealed that most male mice had a significant infiltrate and loss of AT, while the onset of adipose loss in female mice was more variable and the ultimate incidence somewhat lower than that of male mice (Fig. 1 A, right panel). Brown AT showed no infiltrate but resembled WAT due to the aberrant storage of lipids (Fig. S1 A). In addition, the pancreas exhibited signs of metabolic dysregulation with islet hyperplasia, a common phenotype in lipoatrophic mice (Fig. S1 B) (Moitra et al., 1998). Although the body weight of wild-type (WT) and KO male mice remained similar with age, and that of female mice was somewhat increased, the fat mass of both male and female KO mice declined over the study period, while the lean mass of both increased (Fig. 1 B). Notably, the liver mass was increased in KO mice and KO mice exhibited elevated liver triglycerides and significant lipid storage that was evident upon H&E staining (Fig. 1 C). The inability of AT to store triglycerides leads to increased lipid accumulations in all insulin-sensitive non-AT, including the liver. This results in insulin resistance, increased circulating insulin, and the stimulation of lipogenesis in hepatocytes, which contributes to hepatic steatosis in lipodystrophic mice (Shimomura et al., 1998; Shimomura et al., 2000). Analysis at the end of the study period showed that at steady state, both female and male KO mice had elevated blood glucose and elevated insulin (Fig. 1 D). Decreased levels of leptin in male KO mice were observed due to the extreme loss of adipocytes (Fig. 1 D). Finally, insulin tolerance tests indicated that KO mice were insulin-resistant (Fig. 1 E). While the KO mice clearly display signs of altered metabolic homeostasis, similar to other models of Treg dysfunction in the AT (Li et al., 2020; Yu et al., 2022), this model appears to be distinct as the metabolic dysfunction is likely secondary to the extensive inflammatory response and profound loss of adipocytes resulting in redistribution of lipid to the liver.

**Figure S2.**
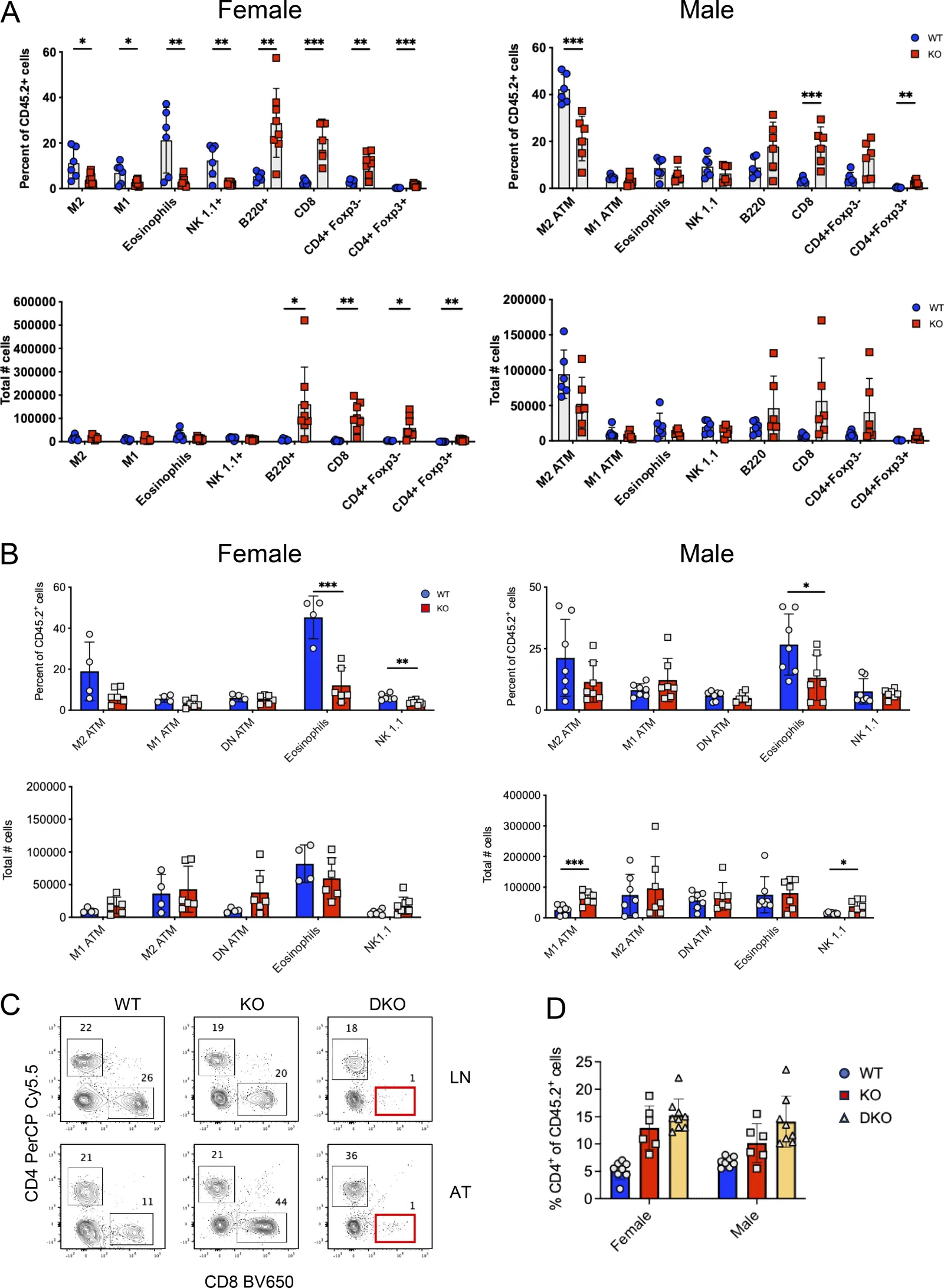
**Mice with Helios-deficient Tregs develop CD8** ^**+**^ **T cell–mediated acquired lipodystrophy. (A)** Subcutaneous AT from 12-wk-old mice was analyzed by flow cytometry for the indicated cell populations. Cell populations are expressed as the percentage of total CD45.2^+^ cells (top) or as the total number of cells (bottom). WT female, *n* = 6; WT male, *n* = 6; KO female, *n* = 8; KO male, *n* = 6. **(B)** Perigonadal AT from 12-wk-old mice was analyzed by flow cytometry for the indicated cell populations. Cell populations are expressed as the percentage of total CD45.2^+^ cells (top) or as the total number of cells (bottom). WT female, *n* = 4; WT male, *n* = 7; KO female, *n* = 6; KO male, *n* = 7. **(C)** CD45.2^+^ F4/80^−^ AT cells and LN cells from 4-mo-old KO and 6-mo-old WT and dKO mice were analyzed for CD4 and CD8 expression. **(D)** Summary data of CD4 expression as a percentage of total CD45.2^+^ AT cells from C. Data are pooled from two independent experiments. WT female, *n* = 8; WT male, *n* = 8; KO female, *n* = 6; KO male, *n* = 6; dKO female, *n* = 9; dKO male, *n* = 8. *P < 0.05, **P < 0.01, ***P < 0.001 by unpaired *t* tests.

**Figure 2.**
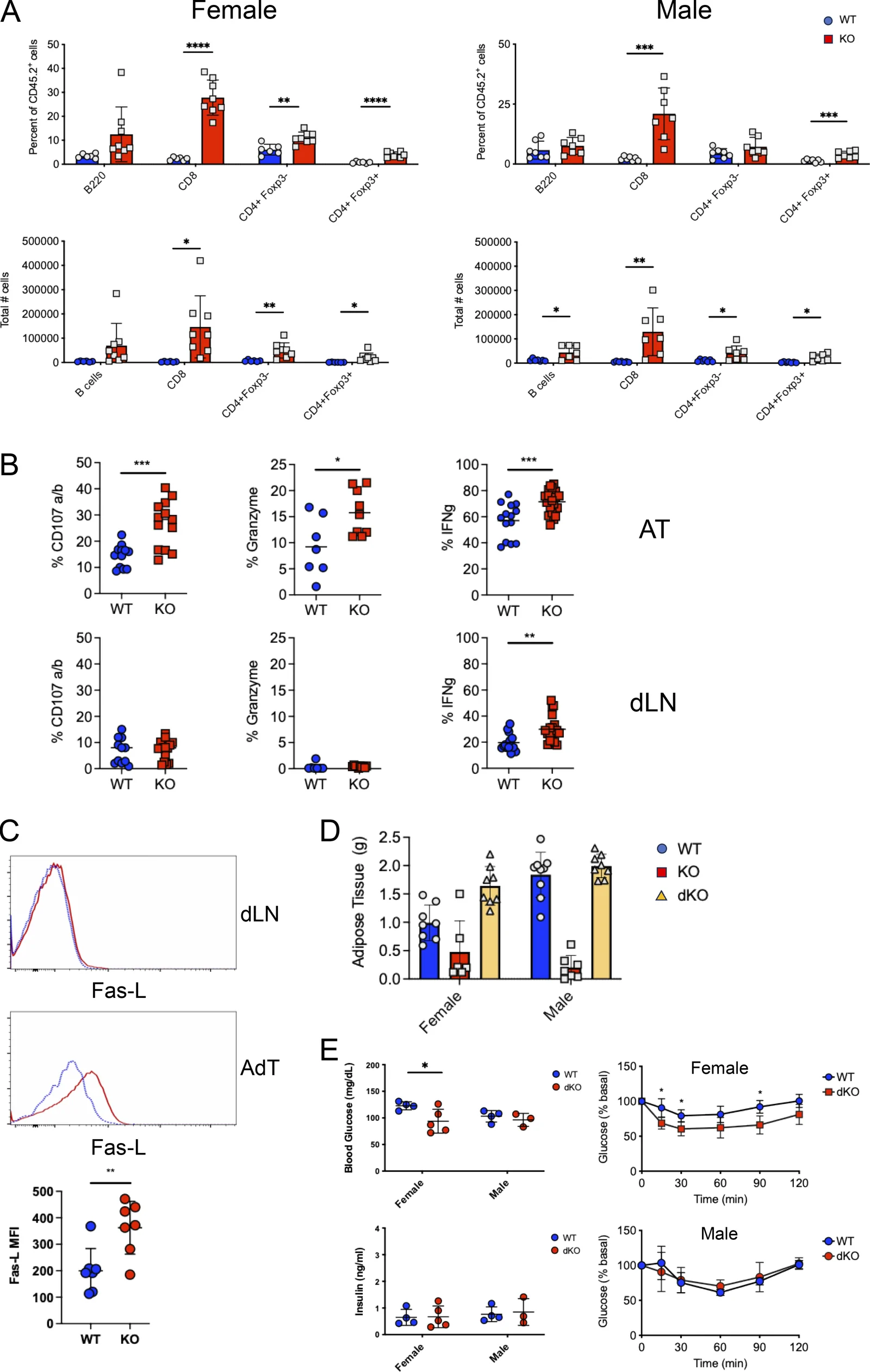
**Mice with Helios-deficient Tregs develop CD8** ^**+**^ **T cell–mediated acquired lipodystrophy. (A)** Perigonadal AT from 12-wk-old mice was analyzed by flow cytometry for the indicated cell populations. Cell populations are expressed as the percentage of total CD45.2^+^ cells (top) or as the total number of cells (bottom). Data are pooled from four independent experiments. WT female, *n* = 6; WT male, *n* = 7; KO female, *n* = 8; KO male, *n* = 7. **(B)** AT cells and dLN cells from 12- to 14-wk-old WT and KO male mice were stimulated with cell stimulation cocktail for 4 h in the presence of αCD107a/b antibodies and then analyzed by flow cytometry. CD45.2^+^ F4/80^−^ CD8^+^–gated cells were analyzed for CD107, granzyme (WT, *n* = 7; KO, *n* = 10), and IFNγ expression (WT, *n* = 14; KO, *n* = 18). Data are pooled from four independent experiments. **(C)** CD45.2^+^ F4/80^−^ CD8^+^ cells from adipose and dLN were similarly analyzed for Fas-L expression. Data are pooled from three independent experiments. WT, *n* = 6; KO, *n* = 7. **(D)** Perigonadal AT from 4-mo-old KO and 6-mo-old WT female, *n* = 8; WT male, *n* = 9; KO female, *n* = 6; KO male, *n* = 7; dKO female, *n* = 8; dKO male, *n* = 8. **(E)** Blood glucose and insulin levels were measured. Blood was collected from the tail vein of fasted female and male WT and dKO mice. In addition, female and male WT and KO mice were fasted overnight, given insulin i.v., and monitored for blood glucose levels at the indicated time points. Data are plotted as the percentage of basal glucose. WT female, *n* = 4; WT male, *n* = 4; KO female, *n* = 5; KO male, *n* = 3. *P < 0.05, **P < 0.01, ***P < 0.001, ****P < 0.0001 by unpaired *t* tests. dLN, draining lymph node.

We have previously shown that WAT is absent in KO mice, including the subcutaneous and visceral AT (Sebastian et al., 2016). Immunophenotyping of subcutaneous and perigonadal AT, adapted from Bapat et al. (2015), was performed at 12–14 wk of age, the age at which both subcutaneous and perigonadal AT loss became apparent (Fig. S1 C). Immunophenotyping of the subcutaneous AT demonstrated differences between WT and KO mice in the percentages of cells across all cell types analyzed in female mice and between several populations in male mice, notably the CD8^+^ T cells and Tregs (Fig. S2 A). However, only the total numbers of B cells, CD8^+^ T cells, CD4^+^ T cells, and Tregs between WT and KO in female mice were significantly different. Although the lymphoid compartment trended toward an increase in males, the result was not statistically significant due to the extreme variability in cell recovery and percentages in the subcutaneous AT. Immunophenotyping of the perigonadal AT demonstrated a decrease in the percentage of eosinophils, which are important for AT homeostasis via IL-4 secretion (Wu et al., 2011) (Fig. S2 B). However, no differences in the absolute number of eosinophils were noted. In addition, there were no significant differences in the percentages or numbers of AT macrophages (ATMs), or in the ratio of M1:M2 ATMs that are typically observed in other models of disrupted adipose homeostasis (Fig. S2 B). In contrast, we observed a striking increase in T cells, particularly in CD8^+^ T cells, both in total percentages and in absolute numbers in the perigonadal AT (Fig. 2 A). The preponderance of CD8^+^ T cells in the inflammatory infiltrate raised the possibility that the loss of adipocytes was mediated by autoreactive, cytotoxic CD8^+^ T cells. In support of this model, CD8^+^ T cells from AT of KO mice expressed increased levels of CD107, granzyme B, and IFNγ and increased expression of Fas-L (Fig. 2, B and C). As adipocytes express Fas (Wueest et al., 2010), it is likely that the Fas/Fas-L pathway may also play a role in the loss of adipocytes. Notably, the increased cytolytic profile of CD8^+^ T cells in the AT was organ-specific, as CD8^+^ T cells from the inguinal LN (the draining lymph node of subcutaneous AT) of KO mice did not display increased markers of cytolytic activity (Fig. 2 B).

To conclusively demonstrate that the AT destruction was mediated by CD8^+^ T cells, we crossed the KO mice to β2-microglobulin–deficient mice. These mice lack MHC class I molecules and possess few to no CD8^+^ T cells (Zijlstra et al., 1990). We confirmed the loss of CD8^+^ T cells in the LN and AT of the double knockout (dKO) mice (Fig. S2 C). As previously shown, both female and male single KO mice exhibited a significant loss of AT compared with WT mice (Fig. 2 D). However, in the absence of CD8^+^ T cells, the dKO mice maintained AT demonstrating that CD8^+^ T cells were required for the loss of AT. The dKO mice retained the CD4^+^ T cell infiltrate in the AT implying that they played a minimal role in adipocyte destruction, but we cannot rule out the possibility that CD4^+^ T cells are required for CD8 help (Fig. S2 D). Furthermore, the metabolic changes observed in the single KO mice (Fig. 1, D and E) were abrogated in the dKO mice (Fig. 2 E). Both fasting glucose and insulin levels in male dKO mice were comparable to those in WT males, with no evidence of insulin resistance detected. In female dKO mice, fasting insulin remained unchanged, while fasting glucose decreased. Notably, female dKO mice showed no evidence of insulin resistance and, in fact, displayed a slight improvement in insulin sensitivity at several time points. Thus, the lipodystrophy and the metabolic abnormalities observed in the Helios KO mice were dependent upon the infiltration of CD8^+^ T cells. Collectively, the loss of adipocytes in the KO mice is mediated via autoreactive CD8^+^ cells through IFNγ-, cytotoxic granule–, and Fas-L–mediated mechanisms resulting in metabolic disruption.

### Helios-deficient Tregs in AT have an altered phenotype

**Figure 3.**
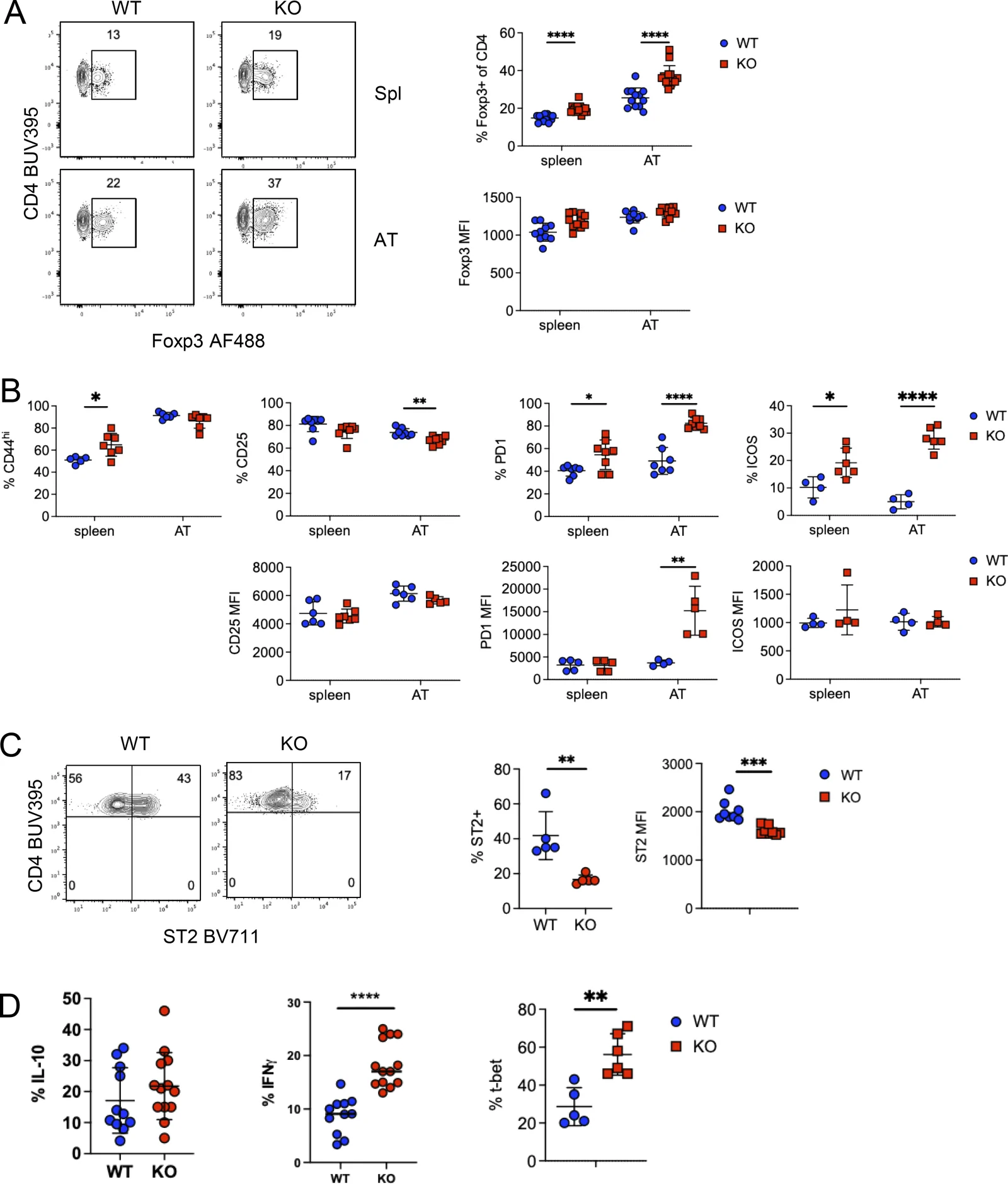
**Helios-deficient AT Tregs have an altered phenotype. (A)** Spleen and AT Tregs from 12- to 14-wk-old WT and KO male mice were analyzed by flow cytometry for Foxp3 expression. Representative plots and summary data of percentages (WT, *n* = 12; KO, *n* = 11) and MFI (WT, *n* = 10; KO, *n* = 11) are shown. **(B–D)** Spleen and AT Foxp3^+^ Tregs from 12- to 14-wk-old WT and KO male mice were analyzed by flow cytometry for CD44 (WT, *n* = 5; KO, *n* = 7), CD25 (WT, *n* = 12; KO, *n* = 8), PD1 (WT, *n* = 7; KO, *n* = 8), and ICOS expression (WT, *n* = 4; KO, *n* = 6), and (C) AT Foxp3^+^ Tregs were analyzed by flow cytometry for ST2 expression (WT, *n* = 5; KO, *n* = 5), ST2 MFI (WT, *n* = 8; KO, *n* = 7), and (D) IL-10 (WT, *n* = 11; KO, *n* = 13), IFNγ (WT, *n* = 11; KO, *n* = 13), and t-bet (WT, *n* = 5; KO, *n* = 6) expression. For all experiments, data are pooled from at least three independent experiments. *P < 0.05, **P < 0.01, ***P < 0.001, ****P < 0.0001 by unpaired *t* tests.

As an initial attempt to determine why Helios-deficient Tregs fail to control autoreactive CD8^+^ T cells, we performed a phenotypic characterization of the AT Tregs. Helios KO Tregs were more numerous as a percentage of total CD4^+^ T cells in both the spleen and the AT, ruling out the possibility of a defect in the migration of Tregs to the AT (Fig. 3 A and Fig. 2 A). In addition, the mean fluorescence intensity (MFI) of Foxp3 in spleen and AT KO Tregs was unchanged, ruling out an effect of Helios on Foxp3 expression. We have previously shown that Tregs in the spleen of 6-mo-old KO mice have a more activated phenotype with increased CD44 expression, ICOS expression, and IFNγ secretion (Sebastian et al., 2016). Analysis of mice at 12–14 wk of age, a time point at which AT was still present, confirmed that CD44 and ICOS expression on splenic KO Tregs was increased (Fig. 3 B). Analysis of CD44 expression on AT Tregs was unrevealing, as CD44 is required for tissue residency and is highly expressed on both WT and KO AT Tregs (Fig. 3 B). CD25 expression on AT Tregs was slightly decreased, indicative of activation. PD-1 expression was moderately increased in splenic KO Tregs and markedly increased in AT KO Tregs. In addition, ICOS was also elevated in AT KO Tregs, again demonstrating that KO Tregs have a more activated phenotype and a more highly activated phenotype in the AT compared with the spleen. Although the percentages of KO Tregs were changed for the indicated markers, only the MFI of PD-1 on KO Tregs in the AT was increased (Fig. 3 B).

It has been shown that the activation of AT Tregs and the expression of the IL-33R (ST2) on Tregs are important for their effector function and their capacity to secrete IL-10 to control metabolic homeostasis in AT (Vasanthakumar et al., 2015). Even though Helios KO Tregs exhibited an activated phenotype, there was a significant decrease in the percentage of KO AT Tregs that expressed ST2 (Fig. 3 C). Moreover, the MFI of ST2 expression was also reduced. However, the percentage of KO Tregs capable of IL-10 secretion was no different from that of WT Tregs, revealing a discordance between the activation state and effector function (Fig. 3 D). Similar to what we previously observed in the spleen (Sebastian et al., 2016), we also noted an increase in Tregs expressing t-bet and capable of secreting IFNγ in the AT of KO mice (Fig. 3 D). This increase is likely secondary to an adaption of the Tregs to the Th1 inflammatory environment but could be secondary to their conversion to a pathogenic phenotype. Thus, in the AT, Helios-deficient Tregs exhibit an activated phenotype and produce IL-10 that is necessary for metabolic homeostasis, but they are deficient in their expression of ST2.

### Transcriptome analysis reveals Helios-deficient Tregs in the AT have an activated phenotype

**Figure 4.**
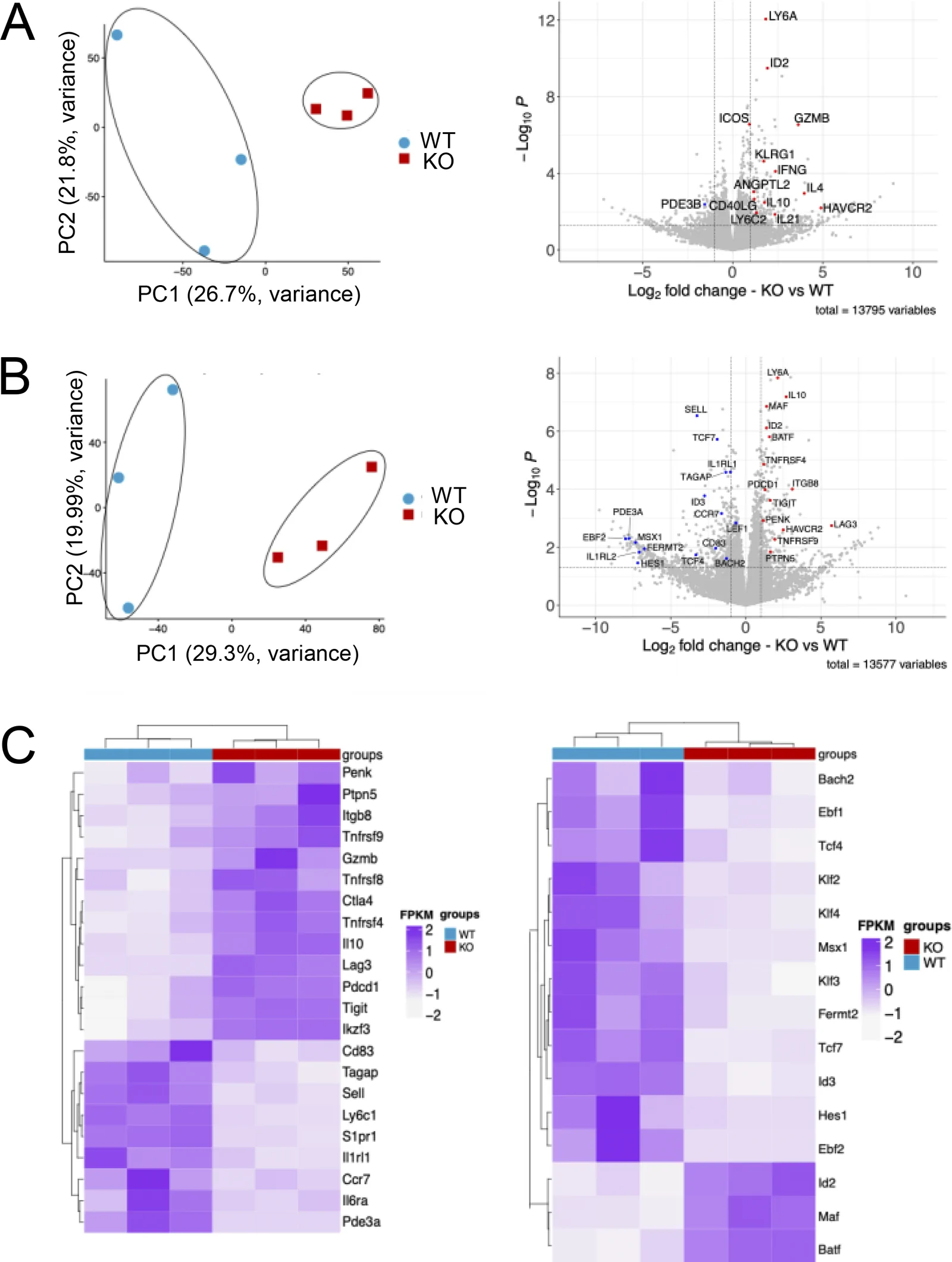
**Helios-deficient AT Tregs have an activated phenotype. (A and B)** WT and KO Tregs were sorted from (A) spleens and (B) AT of 6-wk-old female mice. WT, *n* = 3; KO, *n* = 3. PCA of the transcriptomes of three samples from each phenotype is shown along with a volcano plot comparing the transcriptomes of WT and KO Tregs. **(C)** Heatmaps of selected genes and TFs from B are shown.

To more broadly examine the phenotype of Tregs from KO mice and to determine why Helios-deficient Tregs were dysfunctional, we first performed transcriptome analysis of Tregs from spleen. As the KO mice can exhibit the initial signs of inflammation and T cell activation as early as 8 wk of age, we chose to examine splenic Tregs from WT and KO mice at 6 wk, hoping to reduce or mitigate the effects of activation and inflammation. Principal component analysis (PCA) shows that the WT and KO Tregs cluster independently and a volcano plot of all 13,795 genes that were expressed revealed that even at a young age, 427 genes were differentially expressed (DE), 150 decreased in KO Tregs and 276 genes increased in KO Tregs (Fig. 4 A). One notable DE gene downregulated in KO Tregs was cyclic nucleotide phosphodiesterase 3B (*Pde3B*), which is highly suppressed by Foxp3, and its continued suppression is required for Treg stability (Gavin et al., 2007). We have previously shown that *Pde3b* is more repressed in Helios^+^ tTregs compared with Helios^−^ pTregs, demonstrating that Helios^+^ tTregs are a more activated, stable Treg population (Thornton et al., 2019). Thus, contrary to our expectations, splenic KO Tregs not only maintain downregulated *Pde3b*, but *Pde3b* is reduced compared with WT Tregs, indicating a very stable activated state. Although the splenic Tregs were assayed at 6 wk, prior to the overt manifestations of an autoimmune phenotype, several markers of activation including *Ly6A*, *Ly6c2*, *Havcr2*, *Id2*, *Klrg1*, and *Icos* were increased in the KO Tregs showing that in the absence of Helios, Tregs begin to exhibit an activated phenotype in peripheral lymphoid organs at an early age. In addition, the cytokine genes *Ifnγ*, *Il10*, *Il4*, and *Il21* were also increased in KO Tregs, which should remain suppressed in a stable Treg. Thus, although the splenic KO Tregs exhibit a stable, slightly more activated phenotype, in the absence of Helios they begin to show signs of effector dysregulation.

We next performed transcriptome analysis of AT Tregs, also at 6 wk of age. PCA shows that the WT and KO Tregs cluster independently and a volcano plot of all 13,577 genes revealed significantly more DE genes in the AT compared with the spleen. In AT Tregs, there were 792 DE genes, 321 enriched genes and 470 underrepresented genes in the KO Tregs (Fig. 4 B). Notably, transcriptome analysis of WT and KO Tregs from the AT demonstrated enhanced expression of several of the splenic genes that were increased (*Ly6a*, *Havcr2*, *Id2*, and *Il10*) and additionally many Treg-associated/activation genes (*Itgb8*, *Tnfrsf9*, *Tnfrsf8*, *Ctla4*, *Tnfrsf4*, *Lag3*, *Pdcd1*, and *Tigit*) (Fig. 4, B and C). Furthermore, when the KO AT DE dataset was compared with AnimalTFDB 4.0 (Shen et al., 2023), a database containing TF and TF cofactor genes, it was revealed that 87 TFs were DE by KO Tregs (Table S1). Among the DE TFs, a significant number of TFs or cofactors involved in the restraint of effector T cell activation and differentiation were downregulated in the AT KO Tregs (*Bach2*, *Tcf7*, and *Id3*) and those associated with an activated, effector phenotype (*Id2*, *Maf*, and *Batf*) were enriched in AT KO Tregs (Fig. 4 C). Thus, in the AT, KO Tregs unexpectedly have a highly activated, effector phenotype. In addition, the volcano plot shows a cluster of significantly downregulated genes that are known TFs or cofactors associated with Wnt signaling (*Ebf1*, *Ebf2*, *Msx1*, *Hes1*, and *Fermt2*). Wnt signaling in T cells is important for stem cell generation and restricting terminal differentiation and regulates these processes via Tcf1 and Lef1 (Gattinoni et al., 2009; Jeannet et al., 2010). We also observed a decrease in *Il1rl1*, the gene for ST2, in agreement with decreased ST2 expression shown in Fig. 2 B. However, the decrease in ST2 expression is not consistent with the overall increased activation observed in AT KO Tregs and suggests that AT KO Tregs are highly activated but potentially have defects in terminal differentiation of functional eTregs.

### Helios regulates the key TFs that mediate eTreg differentiation

**Figure 5.**
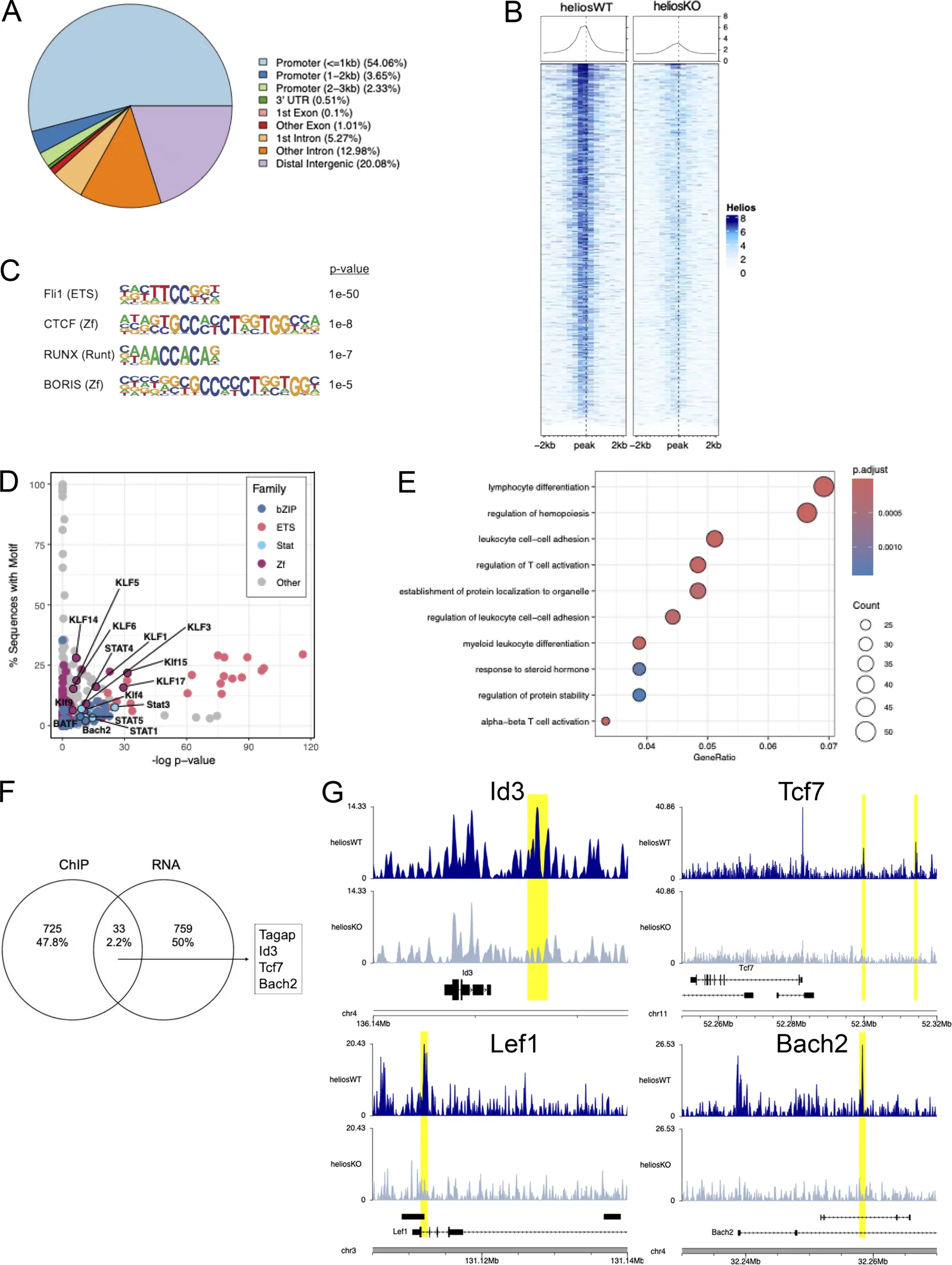
**Helios regulates the key TFs that mediate eTreg differentiation. (A)** Distribution of Helios binding sites in splenic Tregs. **(B)** Average profile plot and corresponding heatmap showing ex vivo Helios ChIP-seq signal from WT and KO Tregs centered on reference Helios peak midpoints. Reference peak coordinates were acquired from CistromeDB (Dataset ID: 57122), representing Helios ChIP-seq data originally generated from splenic Tregs expanded in vitro by Kim et al. (2015). ChIP-seq read density is plotted from −2 kb to +2 kb relative to the peak centers and normalized as RPGC. **(C)** Significant sequence motifs identified by HOMER within Helios binding sites. Displayed motifs represent putative cofactors previously associated with Ikaros (Sun et al., 2022). **(D)** Known motif enrichment identified by Homer at Helios peaks plotted by significance (−log P value) and occurrence (% sequences). Data points are colored by target motif families (bZip, ETS, STAT, and Zf; gray = other). Select significant motifs (KLF, STAT, BATF, Bach2) are denoted with black rings and labeled. **(E)** Gene Ontology Biological Process (GOBP) analysis of enriched Helios binding sites. **(F)** Comparison of ChIP-seq–associated genes and splenic transcriptome DE genes. **(G)** Helios binding sites at selected genes. Note: Apparent enrichments at the Tcf7, Lef1, and Bach2 loci represent narrow PCR artifacts (<100 bp) that did not pass MACS2 peak-calling thresholds. RPGC, reads per genomic content.

**Figure S3.**
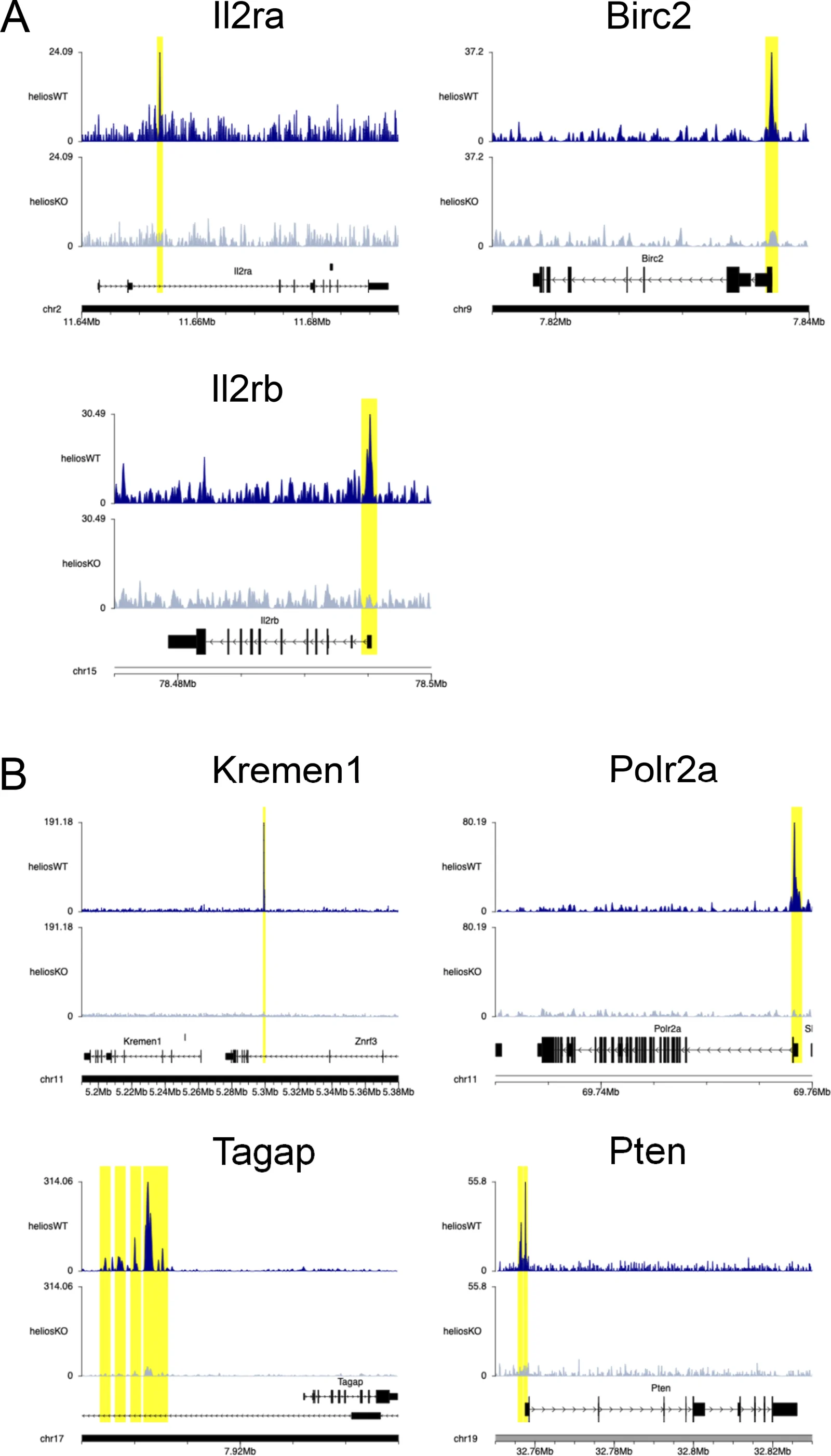
**Helios chromatin binding sites.** Chromatin from Helios^+^ Tregs from Helios-GFP x Foxp3-RFP double reporter mice (WT) and Tregs from Ikzf2^fl/fl^ x CD4-Cre mice (KO) was immunoprecipitated with an anti-Helios antibody. **(A)** Peaks were called based on enrichment relative to KO Tregs, and the y axis indicates RPGC. **(A)** Helios ChIP-seq data for selected loci in common with a previously published dataset. **(B)** ChIP-seq data for additional selected loci. RPGC, reads per genomic content.

Several key TFs control terminal effector differentiation of T cells, including Tregs (Sumida et al., 2024). Id3 inhibits all subsets of eTreg differentiation, while Tcf1, Lef1, and Bach2 inhibit T_H_1-type, T_H_2-type, and T_H_17-type eTregs, but promote T_FR_ differentiation. The downregulation of *Id3*, *Tcf7*, and *Bach2* in AT KO Tregs (Fig. 4 C) raises the possibility that Helios might directly regulate these genes, as well as other TFs involved in eTreg differentiation. To investigate genes potentially regulated by Helios in WT Tregs, we conducted ChIP-seq analysis on splenic Tregs isolated from WT mice. Helios KO Tregs served as a negative control, using the same anti-Helios antibody for the immunoprecipitation. Normalizing WT Tregs to Helios KO Tregs, we identified 986 Helios-enriched binding peaks. Many of these peaks localized to promoter regions of putative target genes (Fig. 5 A). A previously published Helios ChIP-seq dataset generated from in vitro activated and expanded Tregs, distinct from the directly ex vivo Tregs analyzed in the present study, revealed 1,602 target genes (Kim et al., 2015). Despite these methodological differences, a substantial overlap in Helios binding sites was observed between the previously published dataset and the present data generated from WT Tregs (Fig. 5 B). No overlap was observed between the previous dataset using expanded Tregs and the present data from KO Tregs. Representative shared targets included *Il2ra*, *Birc*, and *Il2rb* (Fig. S3 A). To assess similarities in the binding landscapes of Helios and Ikaros, we examined the broader regulatory environment of these regions. Several cofactor binding motifs, previously identified as Ikaros-associated by Sun et al. through Ikaros ChIP-seq, such as Fli1, CTCF, and RUNX, were strongly enriched in our Helios analysis, reflecting highly consistent binding site architectures (Fig. 5 C) (Sun et al., 2022). Additional motif enrichment analysis indicated numerous potential regulatory interactions involving members of the STAT family (STAT1, STAT3, STAT4, and STAT5), the KLF TF family, and the key immune regulators BATF and Bach2 (Fig. 5 D).

Significantly, pathway analysis showed that Helios-bound genes were involved in lymphocyte differentiation, adhesion, and T cell activation (Fig. 5 E). Two of the most enriched and unique DNA regions in our dataset that were bound by Helios were *Kremen1* and *Polr2a*. Kremen1 inhibits Wnt/β-catenin signaling, a pathway crucial for memory formation (Gattinoni et al., 2009; Jeannet et al., 2010), while Polr2 is an RNA polymerase (Fig. S3 B). In addition, Helios bound to multiple regions near *Tagap* and *Pten*. T cell activation Rho GTPase-activating protein has been associated with T cell activation and differentiation (Mao et al., 2004; Tamehiro et al., 2017), and Pten is required to maintain Foxp3 expression and function (Huynh et al., 2015; Shrestha et al., 2015). Overall, this pattern demonstrates that Helios binds to genes involved in activation and differentiation. Overlaying the transcriptome data with the ChIP-seq data revealed 33 overlapping genes (Fig. 5 F and Table S2). Strikingly, Helios bound to chromatin regions in *Id3*, *Tcf7*, *Lef1*, and *Bach2* (Fig. 5 G), all critical TFs for restraining differentiation to eTregs and, as shown for *Id3*, *Tcf7*, and *Bach2*, are decreased at the transcriptional level in KO Tregs (Fig. 4 C). Although a binding peak was observed for *Lef1* and it was transcriptionally decreased in the KO Tregs, it was not included in the common list as it was decreased by 1.6-fold in KO Tregs, below the cutoff of twofold (Fig. 4 B). Thus, Helios transcriptionally controls multiple TFs that inhibit eTreg differentiation, strongly suggesting that in the absence of Helios, the expression of these TFs is diminished, and eTreg differentiation proceeds unchecked.

### Helios-deficient Tregs in AT exhibit an eTreg phenotype

**Figure 6.**
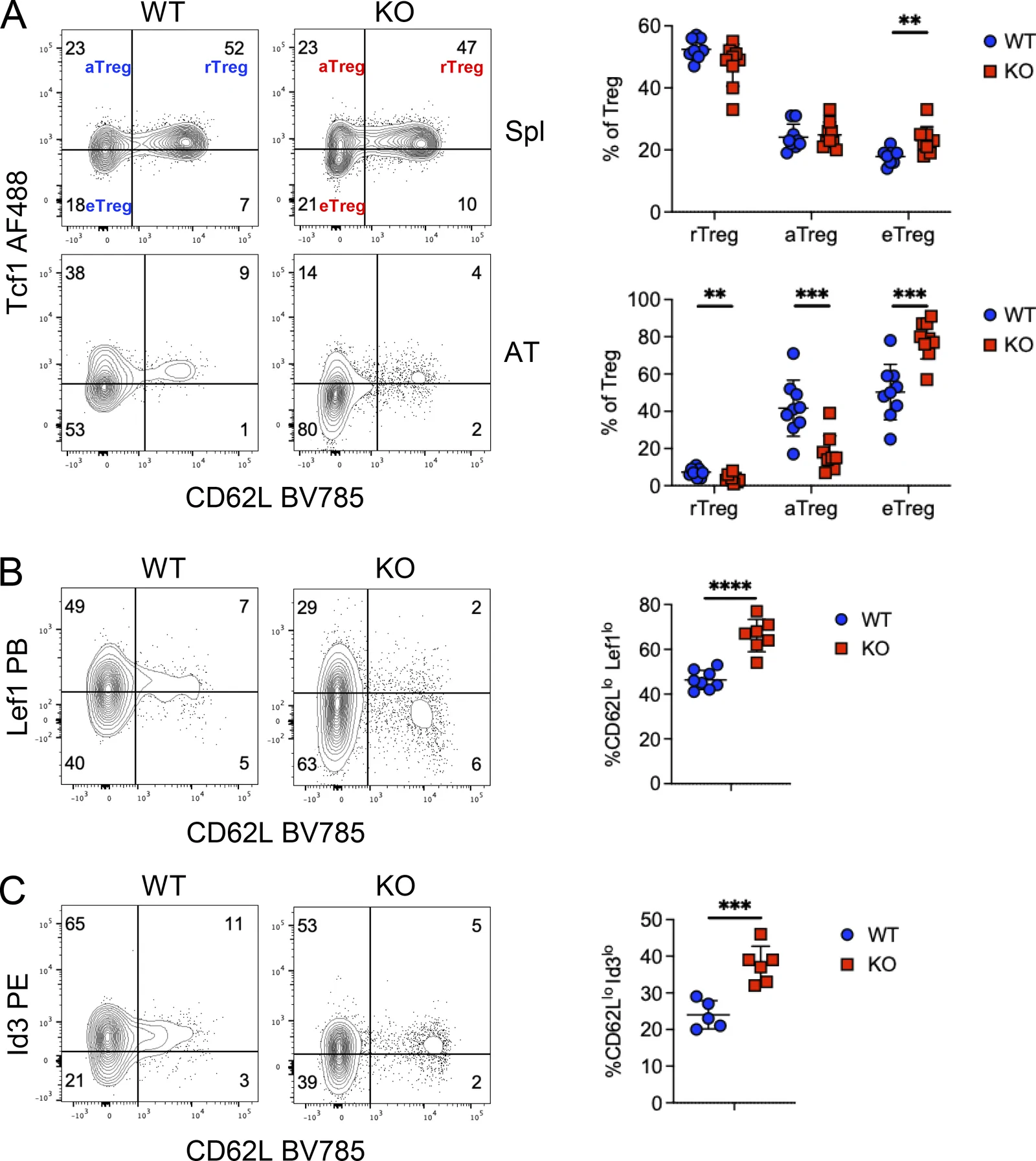
**Helios-deficient Tregs in AT exhibit an enhanced eTreg phenotype. (A)** Foxp3^+^ Tregs from the spleen and AT of 12- to 14-wk-old WT and KO male mice were analyzed by flow cytometry for CD62L and Tcf1 expression. WT, *n* = 8; KO, *n* = 9. **(B and C)** Lef1 expression (WT, *n* = 8; KO, *n* = 7) (B) or Id3 expression (C). WT, *n* = 5; KO, *n* = 6. Data are pooled from at least three independent experiments. **P < 0.01, ***P < 0.001, ****P < 0.0001 by unpaired *t* tests.

Yang et al. (2019) have described three subpopulations of Tregs in peripheral lymphoid organs. rTregs are characterized as CD62L^+^Tcf1^+^, aTregs as CD62L^−^Tcf1^+^, and eTregs as CD62L^−^Tcf1^−^. rTregs become aTregs upon activation, and aTregs then differentiate to eTregs and express markers of terminal differentiation such as Klrg1, IRF4, and Batf. Furthermore, tissue Tregs contain a higher proportion of eTregs compared with spleen. To validate and further explore the transcriptome data that demonstrated a more activated eTreg phenotype in the AT KO Tregs, we performed detailed analysis of TF and cell surface antigen expression by flow cytometry in WT and KO mice. We noted a slight increase in CD62L^−^Tcf1^−^ eTregs in the spleen of KO mice compared with WT, likely reflecting the increased activation and proliferation observed in the inflammatory environment of the periphery (Fig. 6 A). Notably, in the AT, we observed a significant increase in the CD62L^−^Tcf1^−^ eTregs, along with a corresponding decrease of the other two subsets, particularly the CD62L^−^Tcf1^+^ aTreg subset.

It has been demonstrated that *Lef1*, *Id3*, and *Bach2* are also downregulated in eTregs and our transcriptome data indicated that the expression of these genes was lower in AT KO Tregs (Fig. 4 C). Flow cytometry analysis conducted at 12 wk demonstrates that both Lef1 and Id3 were downregulated in AT KO Tregs, corresponding to the decrease in Tcf1, and further demonstrating that the AT KO Tregs have increased eTregs (Fig. 6, B and C). Antibodies to Bach2 are not available, so we were not able to validate the transcriptome data. Thus, in the absence of Helios expression, multiple TFs critical for restraining eTreg differentiation (Tcf1, Lef1, Id3, and, potentially, Bach2) are reduced and Tregs proceed, unchecked, to the eTreg stage.

### Helios-deficient Tregs in AT exhibit a dysfunctional eTreg phenotype

**Figure 7.**
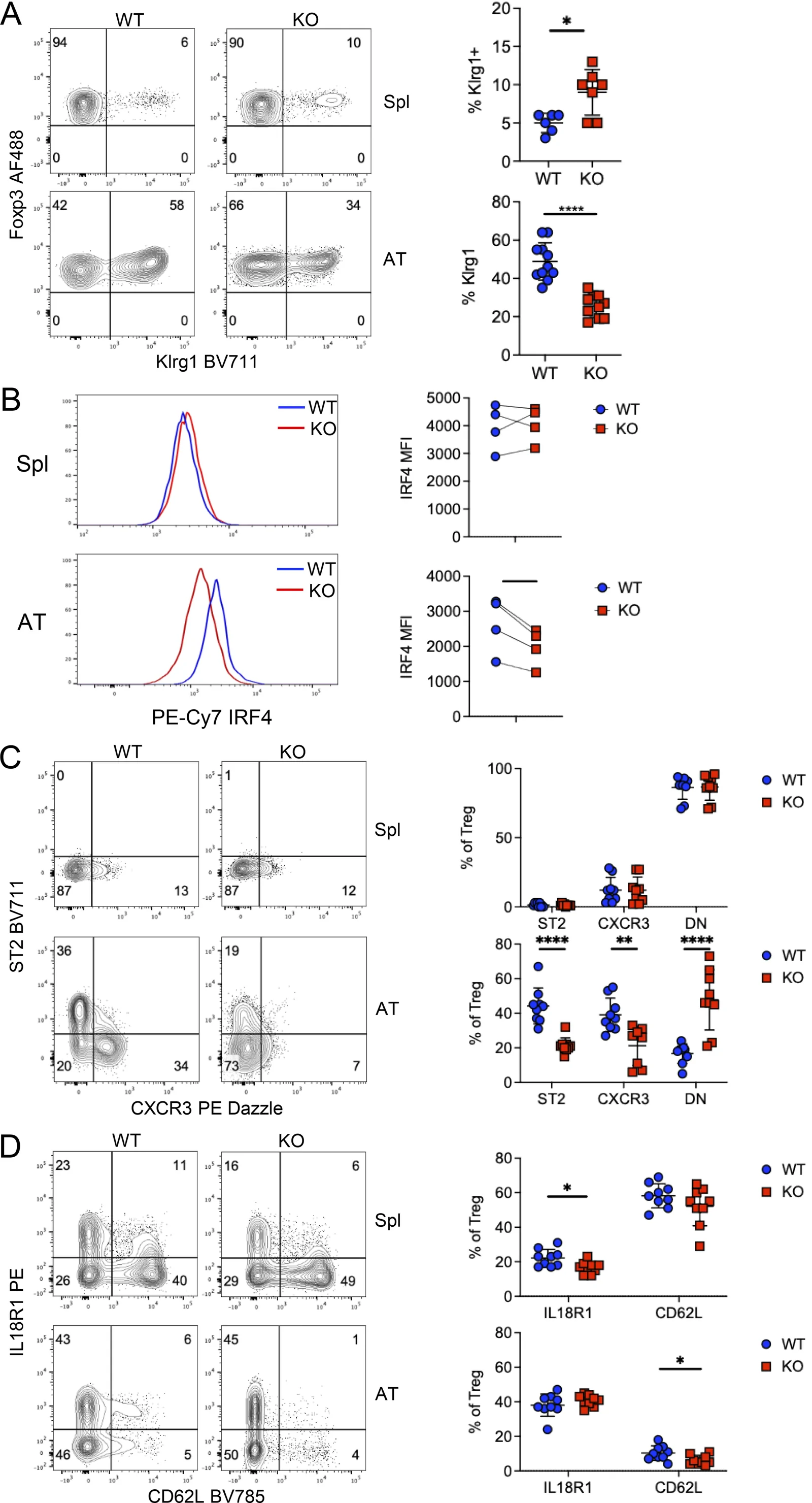
**Helios-deficient AT Tregs lack specific eTreg subsets. (A and B)** Foxp3^+^ Tregs from the spleen and AT of 12- to 14-wk-old WT and KO male mice were analyzed by flow cytometry for (A) Klrg1 expression (WT, *n* = 6; KO, *n* = 7) or (B) IRF4 expression. For IRF4 analysis, four independent experiments with two WT and two KO mice per experiment were performed. WT and KO were averaged and paired for each experiment. **(C and D)** Four experiments were then analyzed with a paired *t* test. Foxp3^+^ Tregs from the spleen and AT of 12- to 14-wk-old WT and KO male mice were analyzed by flow cytometry for (C) ST2 and CXCR3 expression (WT, *n* = 8; KO, *n* = 9) and (D) IL-18R1 and CD62L expression. WT, *n* = 9; KO, *n* = 9. Data are pooled from four independent experiments. *P < 0.05, **P < 0.01, ****P < 0.0001 by unpaired *t* tests.

We next examined Klrg1 and IRF4, markers of terminal differentiation in eTregs. Klrg1 is expressed on terminally differentiated cells, and IRF4 is an important TF for T_H_2-type Tregs and is required for ST2 expression in AT Tregs (Vasanthakumar et al., 2015). Analysis of splenic Tregs showed that Klrg1 was increased in KO Tregs, in agreement with the transcriptome analysis (Fig. 7 A). However, while Klrg1 was expressed at high levels in WT AT Tregs, Klrg1 was significantly decreased in the AT KO Tregs. Moreover, IRF4 expression was also decreased in AT KO Tregs (Fig. 7 B). Thus, while KO Tregs display a proper eTreg phenotype in the periphery, KO Tregs in the AT possess an enhanced ability to differentiate to the eTreg (CD62L^−^Tcf1^−^) stage, but in the absence of Helios fail to express markers of functional terminal differentiation.

Torres et al. have described two distinct subpopulations of eTregs in AT characterized by their expression of either ST2 (Th2-like) or CXCR3 (Th1-like), each with unique functional properties to control adipose homeostasis (Torres et al., 2024). ST2^+^ Tregs in AT have been shown to be necessary for metabolic homeostasis, while CXCR3^+^ Tregs restrain inflammation in AT. To more thoroughly characterize the role of Helios in eTreg terminal differentiation in AT Tregs, we examined these eTreg subset phenotypes by flow cytometry. In the periphery, we have already demonstrated that KO mice fail to generate T_FR_ (Sebastian et al., 2016). Strikingly, we observed that in addition to a substantial decrease in the AT ST2^+^ eTreg population noted in Fig. 3, there was also a profound decrease in the CXCR3^+^ eTreg subset in AT KO Tregs (Fig. 7 C). Concurrently, we observed an increase in ST2/CXCR3 double-negative Tregs. Lastly, Muñoz-Rojas et al. have recently shown that Tregs from epididymal visceral AT (Muñoz-Rojas et al., 2024) can be divided into seven subsets based on single-cell RNA-seq, including quiescent, early activation, *Tbx21*^+^, *Areg*^*hi*^*Il1rl1*^+^, *Areg*^*lo*^*Il1rl1*^+^, *Il18r1*^+^, and *ISG*^+^. The quiescent population was enriched for *Sell* and *Tcf7*, markers typically associated with naïve cells (or rTregs), the early activation population was enriched for *Tnfrsf9* (4-1BB), while the remainder were more clearly effector populations (*Tbx21*^+^ [Th1-like eTregs], *Areg*^*hi*^*Il1rl1*^+^ and *Areg*^*lo*^*Il1rl1*^+^ [ST2^+^ Th2-like eTregs], *Il18r1*^+^ eTregs, and ISG^+^ Tregs, a minor population of eTregs expressing interferon response genes). It remained possible that the absence of Helios diverted AT eTregs from ST2^+^ and CXCR3^+^ populations to other subsets. However, we did not observe the 4-1BB population in either WT or KO AT Tregs, nor did we observe RORγt^+^ T_H_17-type Tregs, or T_FR_ Tregs in the AT. We did detect a significant population of IL-18R1^+^ Tregs in both spleen and AT. While the IL-18R1^+^ KO Treg population was slightly decreased in splenic KO Tregs, it was unchanged in AT KO Tregs (Fig. 7 D). Consequently, the terminal differentiation of the AT KO Tregs did not appear to be diverted to other eTreg subsets. In the absence of Helios, AT Tregs display a pan-eTreg phenotype (CD62L^−^TCF1^−^Lef1^−^), but fail to generate fully differentiated functional eTregs, specifically ST2^+^ eTregs and CXCR3^+^ eTregs that express Klrg1 and IRF4. The marked decrease in the CXCR3^+^ eTreg subset is particularly notable as CXCR3^+^ eTregs also play a unique role in the inhibition of CD8^+^ T cells in the tumor environment (Moreno Ayala et al., 2023). Thus, the loss of CXCR3^+^ eTregs and ST2^+^ eTregs in the AT of KO mice leads to inflammation, cessation of CD8^+^ T cell control, and the ultimate destruction of adipocytes.

### The dysfunctional eTreg phenotype in Helios KO Tregs is cell-intrinsic

**Figure 8.**
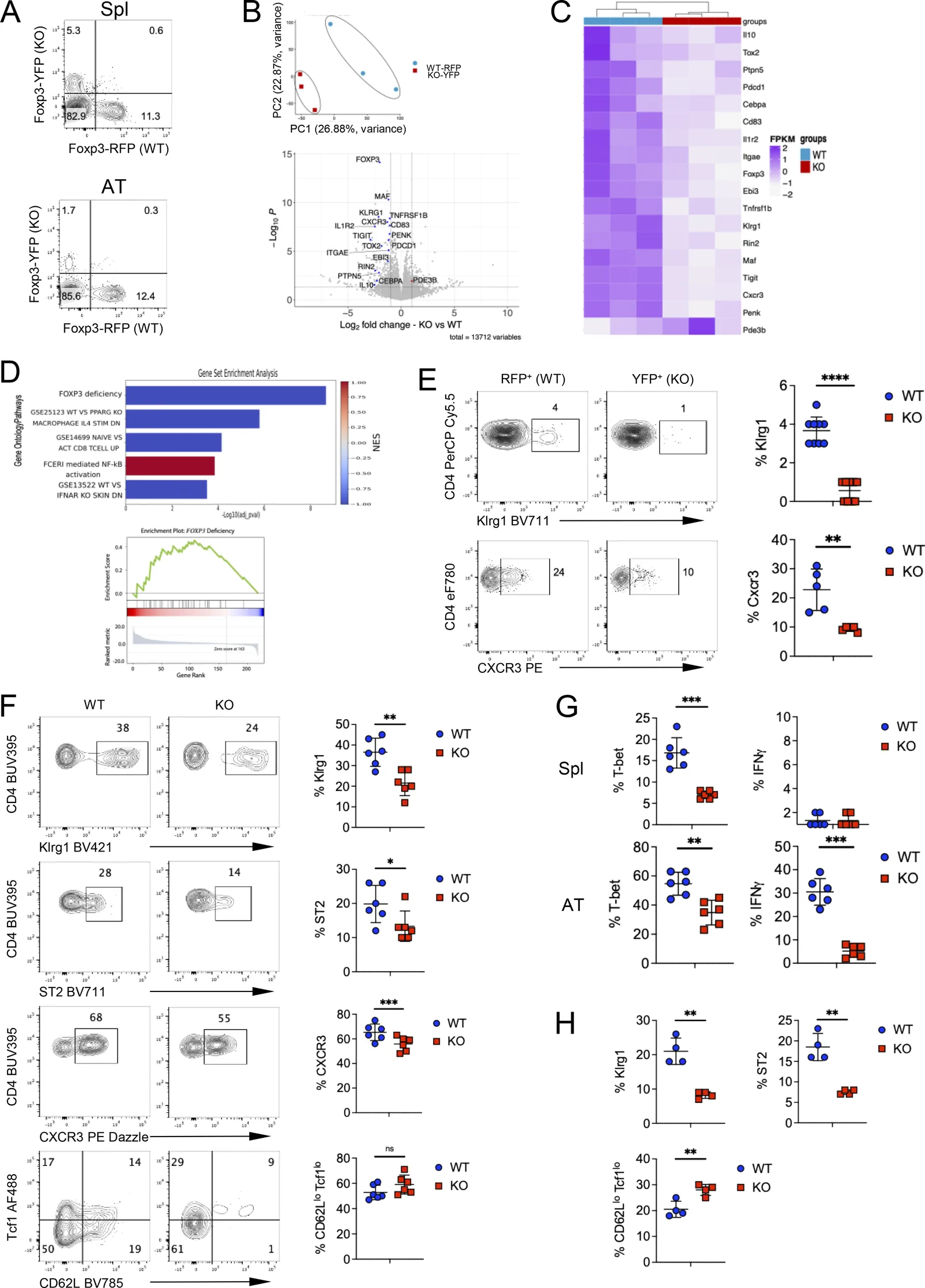
**eTreg functional defect in KO Tregs is cell intrinsic. (A)** CD4^+^ T cells from spleen or AT were analyzed by flow cytometry for expression of RFP (WT) and YFP (KO). **(B)** CD4^+^ RFP^+^ (WT) and CD4^+^ YFP^+^ (KO) Tregs were sorted from spleens of 6-wk-old Ikzf2^fl/fl^ x Foxp3^WT-RFP^/Foxp3^YFP-Cre^ heterozygous female mice. WT, *n* = 3; KO, *n* = 3. PCA of the transcriptomes of three samples from each phenotype is shown with a volcano plot comparing the transcriptomes of RFP^+^ (WT) and YFP^+^ (KO) Tregs with selected activation genes noted. **(C)** Heatmap of selected activation/Treg-associated genes is shown. **(D)** GSEA for DE genes of splenic RFP^+^ (WT) vs. YFP^+^ (KO) Tregs, and GSEA showing the Foxp3 deficiency signature is enriched in the YFP^+^ (KO) Tregs. **(E)** Foxp3^+^ Tregs from the spleen of 8-wk-old Ikzf2^fl/fl^ x Foxp3^WT-RFP^/Foxp3^YFP-Cre^ heterozygous female mice were analyzed by flow cytometry for Klrg1 expression (*n* = 9) and CXCR3 expression (*n* = 5). **(F)** Foxp3^+^ Tregs from AT of 8-wk-old heterozygous female mice were analyzed by flow cytometry for Klrg1, ST2, CXCR3, and CD62L/Tcf1 expression, *n* = 6. **(G)** Foxp3^+^ Tregs from the spleen and AT of 8-wk-old heterozygous female mice were analyzed by flow cytometry for T-bet and IFNγ expression, *n* = 6. **(H)** Foxp3^+^ Tregs from the lungs of 8-wk-old heterozygous female mice were analyzed by flow cytometry for Klrg1, ST2, and CD62L/Tcf1 expression, *n* = 4. For flow cytometry experiments, data are pooled from three independent experiments (E–G) or two independent experiments (H). All data for heterozygous Foxp3 females were analyzed with paired *t* tests. *P < 0.05, **P < 0.01, ***P < 0.001, ****P < 0.0001.

To rule out the possibility that the activation of splenic and AT KO Tregs and the lack of terminally differentiated eTregs in KO AT were due to the extrinsic effects of an inflammatory environment, we also examined Tregs from chimeric Foxp3 heterozygous female mice (*Ikzf2*^*fl/fl*^ x Foxp3^WT-RFP^/Foxp3^YFP-Cre^), where the presence of WT Tregs prevents inflammation. We have previously shown that chimeric mice do not exhibit signs of inflammation or disease but that the YFP (KO) Tregs are at a selective disadvantage compared with the WT Tregs, accounting for only 20% of the splenic Treg population by 6 mo of age. However, in younger mice the ratio in the spleen was only slightly skewed (Sebastian et al., 2016) and (Fig. 8 A). For transcriptome analysis, Tregs were sorted from young heterozygous females at 6 wk. PCA showed that the RFP (WT) and YFP (KO) Tregs cluster independently, and a volcano plot of all 13,712 expressed genes revealed 438 DE genes, 158 genes enriched in KO Tregs and 280 genes decreased compared with WT Tregs (Fig. 8 B). In contrast to the comparison of WT and KO Tregs shown in Fig. 4 A, among the DE genes, *Pde3b* was increased in YFP (KO) Tregs, while *Klrg1* and *Il10* were decreased in YFP (KO) Tregs (Fig. 8 C). In addition, several Treg-associated genes and activation genes such as *Pdcd1*, *CD83*, *Itgae*, and *Tigit*, as well as *Foxp3* itself, were downregulated in the YFP (KO) Tregs. These results indicate a defect in activation of YFP (KO) Tregs in the presence of RFP (WT) Tregs.

To investigate specific pathways that might be influenced by Helios, we performed gene set enrichment analysis (GSEA). Reflecting the decrease in Treg-associated genes observed in the YFP (KO) Tregs, the top enriched pathway was Foxp3 deficiency (Fig. 8 D). Notably, *Klrg1* and *Cxcr3* mRNAs were lower in YFP (KO) Tregs (Fig. 8 C). Cell surface staining confirmed a reduction of Klrg1- and CXCR3-expressing Tregs among the YFP (KO) population, indicating the lack of these terminally differentiated functional eTregs in the absence of Helios is a cell-intrinsic effect (Fig. 8 E).

In contrast to spleen, the ratio of RFP (WT) to YFP (KO) Tregs in the AT of the chimeric Foxp3 heterozygous female mice was highly skewed even at a young age (Fig. 8 A). Thus, the paucity of YFP (KO) Tregs in AT from Foxp3 chimeric mice prohibited transcriptome analysis. However, we were able to analyze the phenotype of the KO Tregs by flow cytometry. Analysis of the AT revealed that Klrg1, ST2, and CXCR3 expression was all reduced in KO Tregs in the chimeric Foxp3 heterozygous female mice (Fig. 8 F). However, while the percentage of CD62L^lo^Tcf1^lo^ eTregs was moderately changed, the increase did not meet statistical significance. We also examined T-bet and IFNγ expression in the chimeric Foxp3 heterozygous female mice. In the spleen, T-bet expression was decreased, but IFNγ expression was not observed. In the AT, we also observed a decrease in T-bet expression in KO Tregs, as well a decrease in IFNγ expression (Fig. 8 G). Thus, in contrast to other markers of terminal differentiation that were cell-intrinsic, increased T-bet and IFNγ expression in the Helios-deficient Treg mice was by cell-extrinsic effects of inflammation.

We have shown that mice with a Treg-specific deletion of Helios develop acquired lipodystrophy, and despite extensive examination, we did not observe immune infiltrate in any other organs. However, ST2 and CXCR3 expression is not unique to AT Tregs. We examined skin and lung Tregs in the Foxp3 heterozygous female mice. In the skin, we did not detect any KO Tregs and the Treg population consisted only of WT Tregs. However, in the lung, as in the AT, the Tregs were skewed toward the WT population, but there were sufficient Tregs for analysis by flow cytometry. When we examined the Tregs from the lung, we observed that like AT, Klrg1 and ST2 expression was decreased in KO Tregs compared with WT Tregs and KO Tregs had increased percentages of eTregs (CD62L^lo^Tcf1^lo^) (Fig. 8 H). Thus, in the competitive environment of the chimera, where WT Tregs can compensate for Helios deficiency, KO Tregs are no longer under extrinsic pressure, revealing an intrinsic role of Helios to maintain an activated state. The reduction of terminally differentiated functional eTregs in the absence of Helios and the absence of inflammation and autoimmune disease in other organs strongly suggests a role of other factors, such as MHC, background genes, and T cell repertoire, in immune homeostasis.

In the tissue environment, Helios is required to directly maintain Id3, Tcf1, Lef1, and Bach2 expression to maintain an aTreg population and regulate eTreg differentiation and it is required during eTreg differentiation to generate terminally functional eTregs. In the absence of Helios, Tregs proceed directly to activated eTregs but with a dysfunctional or exhausted endstage, resulting in a loss of suppressor function.

## Discussion

T cell activation, effector differentiation, and maintenance of a self-renewing pool are complex processes tightly controlled by transcriptional networks. Tregs can be separated into functionally distinct subsets (resting, activated, effector) based on the differential expression of certain key TFs (Sumida et al., 2024). We demonstrate here that the restriction of Treg effector differentiation, mediated through Id3, Tcf1, Lef1, and Bach2, is globally controlled by the TF Helios. Our findings show that this global regulation of differentiation by Helios is critical in the context of tissue inflammation to maintain a sufficient aTreg pool and to generate a functional eTreg pool to prevent autoimmunity.

Treg dysfunction in mice typically results in systemic immune activation and multi-organ leukocyte infiltration and destruction. The organs involved are skin, lungs, liver, colon, salivary glands, and others but, curiously, never AT. It is possible that lipodystrophy is not observed in some models as the mice die at a young age or examination of AT was overlooked. It is unclear why the Helios-deficient mice do not display manifestation of other organ-specific autoimmunity, especially as ST2 and CXCR3 play roles in Treg function in other organs such as skin and lung. We cannot rule out a contribution of mild systemic inflammation in other tissues. In preliminary studies, we have bred the Helios-deficient gene to the BALB/c background. In contrast to C57BL/6 mice, BALB/c mice with Helios-deficient Tregs do show signs of AT loss but die at an early age (10 wk) secondary to autoantibodies to components of the clotting system. Most likely, MHC, background genes, and the TCR repertoire play a role in the development of T effector populations.

While Treg function is assumed to be defective in many models of organ-specific autoimmunity, the number of organ-infiltrating Tregs is frequently paradoxically increased. The development of autoimmune lipodystrophy as the only manifestation of organ-specific autoimmunity in mice with Helios-deficient Tregs afforded us the opportunity to examine in depth the cellular and molecular basis of defects in Treg function at a single tissue site. This model resembles patients with autoimmune disease affecting a single organ system. In general, AT Tregs have been shown to be regulators of adipose metabolism (Becker et al., 2017; Panduro et al., 2016). While the autoimmune lipodystrophy shown here exhibits disordered metabolic regulation, our studies demonstrate that the metabolic dysfunction is likely secondary to the destruction of adipocytes by autoreactive T cells, primarily CD8^+^ T cells. Thus, Tregs not only help to maintain AT metabolic homeostasis but also function in the AT to suppress Th1- and Th2-mediated inflammation and autoimmunity. It should be emphasized that this model of autoimmune lipodystrophy is unusual in that the major pathology is mediated by CD8^+^ T cells. While CD4^+^ T cells are present in the AT infiltrates, they do not appear to mediate adipocyte destruction. In preliminary studies, we have observed that the sera from mice with lipodystrophy contain multiple antibodies to adipocytes, including anti-perilipin-1, which has been observed in patients with autoimmune lipodystrophy (Mandel-Brehm et al., 2023). We have not ruled out the possibility that CD4^+^ T cell helper function is critical for the generation of the CD8^+^ effector cells in the mouse model.

Tcf1, Lef1, Bach2, and Id2/Id3 are the primary core TFs that regulate the development of aTregs while simultaneously restricting eTreg differentiation (Sumida et al., 2024). Mice with deletions of these TFs all exhibit systemic autoimmunity. Miyazaki et al. demonstrated that mice with a Treg-specific deletion of *Id2/Id3* develop a fatal Th2-mediated disease with severe inflammation of the skin and lungs (Miyazaki et al., 2014). Splenic Tregs in these dKO mice had increased eTregs characterized by enhanced expression of CXCR5, PD-1, ICOS, CXCR3, CXCR4, and CTLA-4, but significantly reduced Foxp3 expression. Mice with a germline deletion of *Bach2* also develop a fatal lung disease, and Tregs in these mice express increased percentages of cells with an eTreg phenotype (Tcf1^lo^, Lef1^lo^, with enhanced expression of Klrg1, IRF4, Blimp, T-bet, and Gata3), consistent with a role of Bach2 in the maintenance of T cell quiescence (Kim et al., 2014; Roychoudhuri et al., 2013). Mice with Treg-specific deletion of *Tcf7/Lef1* display systemic immune activation and organ-specific autoimmunity (Xing et al., 2019; Yang et al., 2019).

In contrast to the core TFs, deletion of TFs primarily involved in eTreg differentiation (Batf, IRF4, Gata3, Blimp, t-bet, PPARγ, and Bcl6) displays variable manifestations of autoimmunity in the steady state. IRF4 and Batf bind promoter regions of ST2 and PPARγ, and mice with a deletion of either *IRF4* or *Batf* lack Tregs in AT and also develop systemic autoimmune disease (Delacher et al., 2020; Vasanthakumar et al., 2015). In contrast, deletions of *Gata3* (Wohlfert et al., 2011), Bcl6 (Sawant et al., 2015), *t-bet* (Levine et al., 2017), and *Pparγ* (Cipolletta et al., 2012) result in modest or no autoimmunity, while mice with deletions of *Stat3* (Chaudhry et al., 2009) or *t-bet* and *Gata-3* combined (Yu et al., 2015) present with systemic activation and organ-specific autoimmunity. Among these TFs, PPARγ is uniquely expressed in AT Tregs and not Tregs from peripheral lymphoid organs (Cipolletta et al., 2012). Strikingly, differences in PPARγ expression were not observed between WT and KO AT Tregs, nor were Helios binding peaks detected in the PPARγ locus, demonstrating that Helios does control PPARγ either directly or indirectly. In contrast to the absence of Helios, the lack of PPARγ in Tregs does not result in autoimmunity, highlighting the myriad of Treg functions and the distinct transcriptional programs that regulate these processes.

As Helios globally controls the core TFs that restrain eTreg differentiation, it is quite surprising that Helios KO mice do not exhibit rapid, multi-organ autoimmunity, like that observed in the individual TF-deficient mice. Additional studies are required to determine how Helios fine-tunes the complex expression of these core TFs and the downstream effector TFs and markers.

eTregs in the AT also express two unique markers, ST2 and CXCR3 (Torres et al., 2024). The ST2^+^ and CXCR3^+^ eTreg populations are postulated to play distinct roles in AT homeostasis. ST2^+^ eTregs regulate metabolism, and CXCR3^+^ eTregs regulate inflammation via control of T cell activation. In stark contrast to our studies that demonstrated a marked loss of CXCR3^+^ eTregs and destruction of adipocytes in Helios KO mice, a reduction in CXCR3^+^ AT Tregs via the conditional depletion of *Tbx21* or *Ifng* resulted in the loss of CXCR3^+^ Tregs but did not affect the health or homeostasis of the AT, even though an increase in CD8^+^ T cells was observed. This result is surprising as we observe an increase in T-bet and IFNγ expression in the KO AT Tregs. This result highlights the complex role of Helios in regulating AT homeostasis and demonstrates that Helios regulates a very specific subset of Treg functions.

In contrast to many of the other TFs, Helios is required throughout the entire differentiation process. At the aTreg stage, it directly maintains Id3, Tcf1, Lef1, and Bach2 expression to restrain eTreg differentiation. Notably, in the transcriptome of AT KO Tregs, there is a cluster of genes involved in Wnt signaling that is downregulated (*Msx1*, *FermT2*, *Ebf2*, and *Hes1*). As Wnt signaling is critical for the generation and maintenance of stem cells with self-renewal properties, as well as effector differentiation (Choi et al., 2015; Gattinoni et al., 2009; Jeannet et al., 2010; Notani et al., 2010), it suggests that Helios may play a role in effector differentiation through this pathway. Helios is also required for the generation of functional eTregs that express CXCR3, ST2, Klrg1, and IRF4. Regulation at this step appears to be indirect, as Helios binding sites were not identified within the chromatin of these genes. Motif enrichment analysis revealed potential regulatory interactions between Helios and members of both the STAT and KLF TF families, as well as Bach2 and BATF. Notably, *Klf2*, *Klf3*, and *Klf4* were found to be downregulated in KO Tregs, and the absence of Helios may influence the activity of these potential cofactors. However, further studies are required to clarify how Helios controls the terminal differentiation phenotype.

It is generally accepted that Ikaros family TFs are transcriptional repressors, but our data demonstrate that in Tregs, Helios acts primarily as a transcriptional activator. Helios has, indeed, been shown to be a repressor in both Tregs (Baine et al., 2013) and hematopoietic stem cells to repress megakaryocytes (Cova et al., 2021). However, in cochlear sensory cells, Helios is a transcriptional activator required for outer hair cell (OHC) maturation (Chessum et al., 2018). In its absence, OHC signature genes are reduced and overexpression of Helios in inner hair cells confers an OHC phenotype. Similarly, Helios acts as a transcriptional activator in leukemic stem cells to promote self-renewal genes and is also required to repress myeloid differentiation (Park et al., 2019). Finally, Aiolos and Ikaros have both been shown to act cooperatively with STAT3 as transcriptional activators of *Bcl-6* (Read et al., 2017).

Taken together, the role of Helios in Treg differentiation is multifaceted. Upon appropriate signaling in peripheral lymphoid tissues that leads to T_FR_ differentiation, Helios directly maintains Tcf1 and Lef1 expression and indirectly induces T_FR_-defining genes such as PD-1 and CXCR5. In the absence of Helios, Tcf1 and Lef1 expression is not maintained and T_FR_ are not generated. In the tissue environment, Helios is required to directly maintain Id3, Tcf1, Lef1, and Bach2 expression to inhibit eTreg differentiation. In the absence of Helios, Tregs are not held at the aTreg state, proceed directly to activated eTregs, fail to upregulate Klrg1-, IRF4-, and eTreg-specific markers (ST2/CXCR3), and develop a dysfunctional or exhausted phenotype, with loss of suppressor function. Further analysis of the expression of TFs in Tregs in humans with autoimmunity is clearly warranted and may reveal subtle changes in expression.

## Materials and methods

### Animals

C57BL/6 mice were obtained from Charles River. Foxp3^YFP-Cre^ WT mice were obtained from the Jackson Laboratory. Ikzf2^fl/fl^ and Ikzf2^fl/fl^ x Foxp3^YFP-Cre^ mice have been previously described (Sebastian et al., 2016). Ikzf2^fl/fl^ x Foxp3^RFP^ mice were generated by crossing Ikzf2^fl/fl^ mice and Foxp3-IRES-mRFP mice obtained from the Jackson Laboratory, and Foxp3 heterozygous female mice (Ikzf2^fl/fl^ x Foxp3^WT-RFP^/Foxp3^YFP-Cre^) were generated by crossing Ikzf2^fl/fl^ x Foxp3^YFP-Cre^ mice and Ikzf2^fl/fl^ x Foxp3^RFP^ mice. Beta-2 microglobulin–deficient mice were obtained from the National Institute of Allergy and Infectious Diseases (NIAID)-Taconic contract and were originally obtained from Zijlstra et al. (1990). Helios-GFP x Foxp3-RFP double reporter mice have been previously described (Thornton et al., 2019). Ikzf2^fl/fl^ x CD4-Cre x Foxp3-GFP mice have been previously described (Skadow et al., 2019).

Mouse studies were approved by the Animal Care and Use Committees of NIAID (Animal Study Proposal [APS] LISB 14) and National Institute of Diabetes and Digestive and Kidney Diseases ASP K141-MMC.

### Media, antibodies, and reagents

Mouse cells were cultured in Gibco RPMI 1640 (Thermo Fisher Scientific) supplemented with 10% heat-inactivated FBS, penicillin (100 μg/ml), streptomycin (100 μg/ml), 2 mM L-glutamine, 10 mM HEPES, 0.1 mM nonessential amino acids, 1 mM sodium pyruvate (all from Gibco), and 50 μM 2-ME (MilliporeSigma).

### Histology

Perigonadal visceral AT, brown AT, liver, and pancreas from WT and KO mice were collected, placed in 10% neutral buffered formalin (StatLab), and processed by Histoserv, Inc., for sectioning and H&E staining. Slides were imaged on a NanoZoomer S60 slide scanner and processed on NDP.view2 software (Hamamatsu Photonics). Histological scoring for the AT was done as follows: a score of 1 for moderate localized infiltrate, 2 for moderate disseminated infiltrate, 3 for significant infiltrate and moderate AT loss, 3 for significant infiltrate and significant AT loss, and 5 for complete AT loss.

### Metabolic studies

Body composition (fat mass and fat-free mass) was measured in nonanesthetized mice every other week starting from 6 wk of age by time domain EchoMRI 3-in-1 analyzer (Echo Medical Systems).

For measuring serum metabolites, blood was collected from the tail vein of nonfasted, 30-wk-old mice at 10 am, and allowed to clot for 20 min, and serum was obtained by centrifuging blood samples at 12,000 *g* for 4 min at room temperature. Blood glucose levels were determined using a portable glucometer (Contour Glucometer, Bayer). Leptin (R&D Systems) and insulin (Crystal Chem) were measured by ELISA.

Insulin tolerance test was performed on 34-wk-old mice fasted for 2 h. Insulin (0.75 unit/kg, Humulin R, Eli Lilly) was injected intraperitoneally, and blood glucose was measured using a portable glucometer in blood samples collected from the tail vein at specific time points.

Liver triglyceride was measured by ethanolic KOH hydrolysis followed by a radiometric assay for glycerol as described (Gavrilova et al., 1999).

### Flow cytometry analysis

Single-cell suspensions of peripheral LN (inguinal, axillary, brachial) and spleens were prepared by disruption through a 70-μm nylon filter in HBSS (Gibco) supplemented with 10% heat-inactivated FBS (R&D Systems) and 10 mM HEPES (MilliporeSigma). Red blood cells were lysed with ACK buffer (Gibco). For single-cell suspensions from AT, subcutaneous AT surrounding the peripheral LN or perigonadal VAT was harvested in Fat media (DMEM supplemented with 1% AlbumiNZ Bovine albumin-low free fatty acid [MP Biomedicals] and 50 mM HEPES [Gibco]), minced into small pieces, and then digested in Fat media supplemented with 20 μg/ml DNase I (MilliporeSigma) and 200 μg/ml Liberase TL (Roche) for 20 min at 37°C. The reaction was stopped with Fat media supplemented with 2 mM EDTA, and the samples were passed through a 70-μm nylon filter. Cells were centrifuged to separate lymphocytes (pellet) from adipocytes (floating). For single-cell suspensions from the skin, a 4 × 5 cm rear back skin was harvested, the subcutaneous fat layer was removed, and the upper skin layer and hypodermis were separated. The tissue was finely minced and digested in RPMI supplemented with 10 U/ml DNase I and 250 μg/ml Liberase for 60 min at 37°C. The cells were washed, and the samples were passed through a 40-μm nylon filter. For single-cell suspensions from the lung, the lung was finely minced, and digested in RPMI supplemented with 10 U/ml DNase I and 200 μg/ml Liberase for 30 min at 37°C. The tissue was then processed using the gentleMACS Dissociator (Miltenyi) program m_lung_02.01, and the samples were passed through a 40-μm nylon filter. Finally, the cells were enriched on a 40% Percoll gradient.

All cells were first incubated with TruStain FcX (BioLegend) to block Fc receptors, then stained for surface markers in PBS with 2% FBS. Cells were fixed and permeabilized with Foxp3 TF Staining buffer (Thermo Fisher Scientific) and then stained with intracellular marker antibodies. The following fluorochrome-conjugated antibodies were used: BUV395 anti-CD4 (GK1.5), BUV737 anti-CD8a (53–6.7), BV711 anti-ST2 (ST2), AF488 anti-TCF1 (S33-966), PE-Cy7 anti-IFNγ (XMG1.2), PE anti-ID3 (S30-778), PE anti-T-bet (O4-48), PE anti-CXCR3 (CXCR3-173), PE anti-Siglec-F (E50-2440), and PE anti-B220 (RA3-6B2), all from BD Biosciences; AF700 anti-CD45.2 (104), PerCP Cy5.5, FIRE, or BV421 anti-CD4 (RM4-5), BV650 or BV510 anti-CD8a (53–6.7), BV785 anti-CD62L (MEL-14), PE anti-IL-18R1 (A1707D), PE-Dazzle anti-CXCR3 (CXCR3-173), PE-Cy7 anti-IRF4 (IRF4.3E4), BV711 anti-Klrg1 (2F1/KLRG1), PE-Cy7 anti-PD1 (29F.1A12), BV421 anti-CXCR5 (L138D7), Pacific Blue anti-CD44 (IM7), BV711 anti-CD69 (H1.2F3), BV605 anti-B220 (RA3-6B2), BV711 anti-NK1.1 (PK136), PE anti-CD95L (MFL3), FITC anti-granzyme B (QA16A02), FITC anti-granzyme C (SFC1D8), PE anti-CD107a (1D4B), PE anti-CD107b (M3/84), and Pacific Blue anti-Helios (22F6), all from BioLegend; Pacific Blue anti-Lef1 (C12A5) from Cell Signaling; eFluor or AF488 anti-Foxp3 (FJK-16 s), APC-Fluor 780 anti-TCRβ (H57-597), eFluor 450 anti-CD3(17A2), APC-Cy7 anti-CD11b (M1/70), PerCP Cy5.5 anti-CD11c (N418), PE-Cy7 anti-CD206 (MR6F3), PE-Cy7 anti-granzyme A (GzA-3G8.5), PE anti-IL-10 (JES5-16E3), PE anti-ICOS (7E.17G7), eFluor 780 anti-CD4 (RM4-5), and eFluor 450 anti-NK1.1 (PK136), all from Thermo Fisher Scientific. Dead cells were excluded with Ghost Violet 510 (Cell Signaling) or Zombie Yellow (BioLegend). For CD107 and cytokine staining, cells were plated in complete media and stimulated with Cell Stimulation Cocktail plus Protein Transport Inhibitors (Thermo Fisher Scientific) for 4 h before staining. All flow cytometry experiments were acquired on a custom Symphony A3 or A5 (BD Biosciences) and analyzed with FlowJo version 10.8.1 software (Tree Star).

### Statistics

For flow cytometry, data were plotted and analyzed in GraphPad Prism. For WT and KO comparisons, data were analyzed with unpaired *t* tests. For heterozygous Foxp3 females, data were analyzed with paired *t* tests. *P < 0.05, **P < 0.01, ***P < 0.001, ****P < 0.0001.

### RNA-seq gene expression profiling

Single-cell suspensions were prepared from the pooled spleen of 6- to 7-wk-old Foxp3 heterozygous female mice (Ikzf2^fl/fl^ x Foxp3^WT-RFP^/Foxp3^YFP-Cre^) and from the pooled spleen or pooled AT of 6- to 7-wk-old Foxp3^YFP-Cre^ (WT) and Ikzf2^fl/fl^ x Foxp3^YFP-Cre^ (KO) mice. Using a dump gate for CD8, NK, and B cells, Tregs were isolated by FACS on a BD FACSAria (BD Biosciences).

### RNA extractions

Sorted mouse Tregs were harvested into 1 ml of TRIzol. 800 μl from each sample was then taken, and 200 μl of fresh TRIzol and 200 μl 1-bromo-3-chloropropane (MilliporeSigma) were added; the samples were mixed, incubated for 5 min, and centrifuged for 15 min at 16,000 × *g* at 4°C. RNA was extracted using Agencourt RNAdvance Tissue Kit (Beckman Coulter) per the manufacturer’s instructions (Protocol 000473v003). The RNA quality was assessed using Agilent 2100 Bioanalyzer using RNA 6000 Pico Kit (Agilent Technologies). RNA quantity was measured using a RiboGreen fluorescence assay (Thermo Fisher Scientific) on a Tecan Spark (Tecan).

### RNA-seq and data analysis

Sequencing libraries were prepared using the SMART-seq mRNA LP (with UMIs) kit (Takara Bio USA) per the manufacturer’s instructions with an input amount of 800 pg of total RNA per sample. The library quality was assessed using the Agilent 2100 Bioanalyzer High Sensitivity kit (Agilent Technologies). Libraries were quantified using the Qubit Fluorometer dsDNA High Sensitivity kit (Thermo Fisher Scientific), and pooled to an equal molar ratio to a final loading concentration of 800 pM. Libraries were sequenced on a NextSeq 2000 using a P3 200 cycle reagent kit (Illumina) as paired-end 2 × 100 base pair reads.

Raw RNA-seq reads were processed with RNA-seek pipeline (https://github.com/skchronicles/RNA-seek). Normalized reads were subsequently used for differential expression analysis with eVITTA version 1.3.1. DE genes were visualized using heatmap and volcano plots, with significance defined as nonmultiple testing-adjusted P value of > 0.05 and an absolute fold change >1. All plots were generated using R version 4.5.1.

GSEA was performed using iDEP (Ge et al., 2018). GSEA on spleen samples was performed with the gene set libraries Reactome, KEGG, and Enrichr, using a false discovery rate cutoff of 0.01 and a minimum gene set size of 5. Plots were generated with Python 3.13.5. Term enrichment bar plots are made with the matplotlib package, version 3.10.0, selecting the top five terms by adjusted P value. GSEA plots were made with the gseapy package, version 1.3.0 (Fang et al., 2023).

### ChIP, ChIP-seq, and data analysis

Splenic CD4^+^Foxp3^+^Helios^+^ Tregs were sorted from male and female Helios-GFP x Foxp3-RFP mice (WT), and splenic CD4^+^Foxp3^+^ Tregs were sorted from male and female Ikzf2^fl/fl^ x CD4-Cre x Foxp3-GFP mice (KO) as negative controls. The cells were cross-linked in 1% formaldehyde in media for 10 min at room temperature and sonicated in shearing buffer (0.4% SDS in TE buffer) to generate 100- to 400-bp DNA fragments. DNA–protein complexes were immunoprecipitated with Helios rabbit mAb (D8W4X) (Cell Signaling) and protein A–coated magnetic beads (10001D) (Invitrogen). Purified DNA fragments were ligated to indexed adapters, and ChIP-seq libraries were generated with BioLabs library prep kits (NEB#E7546S/L and NEB#E7595S/L).

Sequencing was performed by the NCI Advanced Technology Research Facility. Libraries were pooled and sequenced on NextSeq 2000 P2 using paired-end sequencing.

ChIP-seq processing. Reads were processed using the chrom-seek (1.1.0) pipeline (https://github.com/OpenOmics/chrom-seek). Reads were trimmed with Cutadapt version 4.4 and then aligned to mm10 GRCm38.p6 using BWA version 0.7.17. All reads aligning to the mm10 version 2 blacklist regions were identified and removed with Picard SamToFastq (https://broadinstitute.github.io/picard/). Reads with a mapQ score <6 were removed with SAMtools version 1.17, and PCR duplicates were removed with Picard MarkDuplicates. Data were converted into bigwigs for viewing and normalized by reads per genomic content using deepTools version 3.5.1. To evaluate signal enrichment, reference peak coordinates for Helios binding in murine Tregs (CistromeDB: 57122) were utilized, representing data originally generated by Kim et al. (GEO accession no. GSM1876372). Coverage plots were created using karyoploteR version 1.34.2 and GenomicRanges. Narrow peaks were called using macs version 2.2.7.1 treating matching KO samples as controls. Enriched motifs were identified using the homer version 4.11.1 function findMotifsGenome.pl with size “given.” Peaks were associated with features using ChIPseeker version 1.44.0 and annotated using Uropa version 4.0.2 and the GENCODE reference M18. For Uropa, peaks were associated with the nearest protein-coding gene’s TSS. Pathway enrichment (GO Biological Processes) was accomplished using clusterProfiler version 4.14.6, and related significant pathways were collapsed using a 0.7 cutoff. Plotted pathways were chosen from the top 15 with the most significant adjusted P values. This work utilized the computational resources of the National Institutes of Health High Performance Computing Biowulf cluster (https://hpc.nih.gov).

### Online supplemental material

Fig. S1 shows supporting data for Figs. 1 and 2, including additional histology of brown AT and pancreas and the gating strategies for AT immunophenotyping by flow cytometry. Fig. S2 shows supporting data for Fig. 2, including immunophenotyping of subcutaneous AT and perigonadal AT from WT and KO mice. Fig. S2 also includes controls to demonstrate CD8^+^ T cell deletion in dKO mice and quantitation of adipose CD4^+^ T cells in WT, KO, and dKO mice. Fig. S3 shows supporting data for Fig. 5, including ChIP-seq data showing Helios binding sites at additional genes. Table S1 shows supporting data for Fig. 4 and contains a list of TF genes DE in adipose Tregs from KO mice. Table S2 shows supporting data for Fig. 5 and contains a list of transcripts in common between the RNA-seq and ChIP-seq datasets.

## Supplementary Material

Table S1 shows a list of TFs in the AT transcriptome dataset.

Table S2 shows a list of common genes between ChIP-seq and AT RNA-seq.

## Data Availability

The mouse lines generated in this study are available from the Shevach lab with a material transfer agreement. The data underlying all figures are available in the published article and its online supplemental material. The underlying RNA-seq data for Figs. 3 and 7 have been deposited at GEO accession number GSE330646, and the underlying ChIP-seq data for Fig. 4 have been deposited at GEO accession number GSE330583.
