# Supplementary material for: Helios is critical for Treg effector differentiation in adipose autoimmunity
Source: J Exp Med. 2026 Oct 1;223(11):e20260870. doi: 10.1084/jem.20260870 (PMC13629518; doi:10.1084/jem.20260870)
Supplement: Supplementary file 1 [file jem_20260870_tables1.docx]

Table S1. List of Transcription Factors in AT Transcriptome Dataset

| 2010315B03RIK | HDAC10 | MAGED1 | TRIB3 |
| --- | --- | --- | --- |
| 4930522L14RIK | HDAC9 | MEF2C | TSC22D1 |
| APEX1 | HES1 | MEIS1 | ZBTB10 |
| ARNT2 | HIC2 | MSX1 | ZBTB20 |
| ATF3 | HIF1A | MXD1 | ZEB2 |
| AU041133 | HOXD8 | NELFE | ZFP24 |
| BACH2 | ID2 | NFIB | ZFP267 |
| BATF | ID3 | NOTCH1 | ZFP35 |
| BAZ1A | IFI211 | NR4A2 | ZFP426 |
| BAZ2B | IKZF3 | PADI2 | ZFP455 |
| BCL3 | IRF8 | PDLIM1 | ZFP579 |
| CAVIN1 | JDP2 | PHF10 | ZFP595 |
| DBP | KDM6B | PLEK | ZFP617 |
| DTX1 | KLF11 | RBPJ | ZFP689 |
| E2F2 | KLF2 | RUNX2 | ZFP708 |
| E2F3 | KLF3 | SMAD1 | ZFP808 |
| EBF1 | KLF4 | SNAPC4 | ZFP93 |
| EBF2 | KLF9 | SOX18 | ZFP948 |
| EGR1 | KMT2D | SSBP2 | ZFP952 |
| EGR2 | LITAF | TCF7 | ZHX2 |
| EPAS1 | MAF | TCF7L1 |  |
| GM6712 | MAFG | THRA |  |
