# Supplementary material for: Helios is critical for Treg effector differentiation in adipose autoimmunity
Source: J Exp Med. 2026 Oct 1;223(11):e20260870. doi: 10.1084/jem.20260870 (PMC13629518; doi:10.1084/jem.20260870)
Supplement: Supplementary file 2 [file jem_20260870_tables2.docx]

Table S2. List of common genes between ChIPSeq and AT RNA Seq

| BACH2 | ID3 | PTRHD1 |
| --- | --- | --- |
| BCL3 | IFNGR2 | RASGRP2 |
| C2CD2 | IKZF3 | RGCC |
| CCR5 | IL6RA | SEERPINI1 |
| CD83 | KIFAP3 | SFXN2 |
| DDIT4 | KLF9 | SKAP2 |
| DTX1 | KMT2D | SNRNP35 |
| EGR1 | LMNA | TAGAP |
| EGR2 | MRPL45 | TCF7 |
| GRB7 | PDK1 | WARS2 |
| HOXD8 | PLEKHA1 | ZFP617 |
